# *SNCA* genetic lowering reveals differential cognitive function of alpha-synuclein dependent on sex

**DOI:** 10.1186/s40478-022-01480-y

**Published:** 2022-12-14

**Authors:** Jennifer L. Brown, Damyan W. Hart, Gabriel E. Boyle, Taylor G. Brown, Michael LaCroix, Andrés M. Baraibar, Ross Pelzel, Minwoo Kim, Mathew A. Sherman, Samuel Boes, Michelle Sung, Tracy Cole, Michael K. Lee, Alfonso Araque, Sylvain E. Lesné

**Affiliations:** 1 Graduate Program in Neuroscience, University of Minnesota, Minneapolis, MN USA; 2 Department of Neuroscience, University of Minnesota, Minneapolis, MN USA; 3 Medical Scientist Training Program, University of Minnesota, Minneapolis, MN USA; 4 Institute for Translational Neuroscience, University of Minnesota, Wallin Medical Biosciences Building (Room 4-114), 2101 Sixth Street SE, CDC 2641, Minneapolis, MN 55414 USA; 5 Ionis Pharmaceuticals Inc., Carlsbad, CA USA; 6 Present Address: Medical Scientist Training Program, University of Texas Southwestern Medical School, Dallas, TX 75390 USA; 7 Present Address: Graduate Program in Molecular and Cellular Biology, University of Washington, Seattle, WA 98195 USA; 8 Present Address: Department of Neurosciences, University of the Basque Country UPV/EHU, Leioa, Spain; 9 Present Address: Bloomberg School of Public Health, Johns Hopkins University, Baltimore, MD 21218 USA; 10 Present Address: n-Lorem Foundation, Carlsbad, CA 92010 USA

**Keywords:** Alpha-synuclein, Spatial memory, Sex, Antisense oligonucleotide, Synucleinopathy, Alzheimer’s disease, Early growth response 1

## Abstract

Antisense oligonucleotide (ASO) therapy for neurological disease has been successful in clinical settings and its potential has generated hope for Alzheimer’s disease (AD). We previously described that ablating *SNCA* encoding for α-synuclein (αSyn) in a mouse model of AD was beneficial. Here, we sought to demonstrate whether transient reduction of αSyn expression using ASO^*SNCA*^ could be therapeutic in a mouse model of AD. The efficacy of the ASO^*SNCA*^ was measured via immunocytochemistry, RT-qPCR and western blotting. To assess spatial learning and memory, ASO^*SNCA*^ or PBS-injected APP and non-transgenic (NTG) mice, and separate groups of *SNCA*-null mice, were tested on the Barnes circular maze. Hippocampal slice electrophysiology and transcriptomic profiling were used to explore synaptic function and differential gene expression between groups. Reduction of *SNCA* transcripts alleviated cognitive deficits in male transgenic animals, but surprisingly, not in females. To determine the functional cause of this differential effect, we assessed memory function in *SNCA*-null mice. Learning and memory were intact in male mice but impaired in female animals, revealing that the role of αSyn on cognitive function is sex-specific. Transcriptional analyses identified a differentially expressed gene network centered around *EGR1*, a central modulator of learning and memory, in the hippocampi of *SNCA*-null mice. Thus, these novel results demonstrate that the function of αSyn on memory differs between male and female brains.

**Supplementary Information:**

The online version contains supplementary material available at 10.1186/s40478-022-01480-y.

## Introduction

The number of people suffering from Alzheimer’s disease (AD) continues to grow, and the treatment options remain few. AD is a progressive neurodegenerative disease characterized by a loss of cognitive function, including reasoning and memory. The hallmarks of AD, tau neurofibrillary tangles and amyloid-beta (Aβ) plaques, are rarely seen in isolation. Over 50% of AD brains also display Lewy bodies, composed of aggregated alpha-synuclein (αSyn), which are classically linked to Parkinson’s disease [48, 52]. While the appearance of mixed proteinopathies is now more commonly recognized in the aging brain [8], accumulating evidence suggests a link between αSyn and AD [25, 29, 30, 38].

Transgenic mouse models have been used to investigate the potential contribution of αSyn to AD phenotypes but these studies have often resulted in diverging conclusions [3, 11, 35, 49, 56]. Using a bidirectional approach in a controlled genetic background, data from our lab showed that male αSyn knock-out mice overexpressing human mutant amyloid precursor protein (APP) displayed improved spatial reference memory and survival but increased Aβ deposition [25]. Conversely, male mice overexpressing human wild-type αSyn and APP presented with an exacerbated cognitive deficit accompanied by a decrease in Aβ plaques [25]. These results indicated that αSyn is a profound modulator of AD phenotypes in male APP mice. Consequently, evidence in both humans and mice suggests that decreasing αSyn might be beneficial in AD.

Because our past work utilized a genetic approach rather than a method suitable for the clinic, we initially aimed to investigate the use of αSyn-lowering antisense oligonucleotides as a possible translational therapy for AD. Briefly, antisense oligonucleotides (ASOs) are short, single-stranded DNA sequences which can be utilized to reduce the expression of target gene mRNA and protein in many human disease models [27, 47], including AD [14]. The Food and Drug Administration has approved Spinraza^®^ (Nusinersen), an ASO therapy for adults and infants suffering from spinal muscular atrophy, demonstrating that ASOs can be safe and effective treatments for some neurological disorders [5]. Consequently, the interest in ASO-mediated translational approaches has skyrocketed in this field [47] and generated hope in patients and their families.

In this study, we first used a single injection of an ASO targeting mouse *SNCA* (ASO^*SNCA*^), the gene that encodes αSyn, to reduce αSyn mRNA and protein in a well-known APP transgenic mouse model of AD and non-transgenic (NTG) littermates. While the male APP mice treated with the ASO^*SNCA*^ showed cognitive improvements, both APP and NTG female mice unexpectedly performed worse. To determine the functional cause of this sex-specific effect, we performed behavioral and transcriptional analyses using αSyn knockout (αSyn-KO) mice. Learning and memory studies revealed that *SNCA* gene deletion caused a cognitive delay in female animals only. Transcriptomic profiling identified a gene network centered around *EGR1*, a key transcription factor involved in learning and memory [24, 60], that was differentially expressed across sex in the hippocampi of αSyn-KO mice. Finally, we confirmed sex-dependent changes in Egr1 protein expression in hippocampal neurons from αSyn-KO mice. To our knowledge, these results are the first to reveal a sex-specific role of αSyn on cognitive function and transcriptional responses.

## Materials and methods

### Animals

Two genetically-modified lines were used: (i) *SNCA*-null mice [1] and (iii) J20 APP (originally called hAPPJ20) mice [39]. *SNCA*-null mice were obtained from Jackson laboratories and backcrossed to C57BL6/J for greater than 10 generations. Every 6 months, the homozygous KO mice are outbred to wild-type C57BL6/J and homozygous KO mice are reconstituted from the mating of heterozygous animals. Both male and female animals were used in equal numbers for biochemical studies and Barnes maze behavioral testing. All animal procedures and studies were reviewed and approved by the University of Minnesota Institutional Animal Care and Use Committee and Institutional Review Board and the animals' care was in accordance with institutional guidelines.

### Antisense oligonucleotides

The synthesis and purification of all lyophilized ASOs was formulated in PBS as previously described [12] and stored at –20 °C (kind gifts from Drs. Tracy Cole and Holly Kordasiewicz, Ionis Pharmaceuticals Inc., Carlsbad, CA, USA). The sequences for ASO1 and ASO2 (5′-3′) are TTTAATTACTTCCACCA and CTGTTAAGTCACAAGCA respectively. The ASOs contain modified nucleotides, including 2′-O-methoxyethylribose (MOE) and (S)-2′,4′-constrained 2′-O-ethyl (cEt) 2′-O-methoxyethyl modifications, as well as phosphorothioate backbone modifications.

### Primary neurons

Primary neuron cultures were prepared as previously described [40, 54].

### Protein extractions

Soluble aggregation-prone protein levels in brain tissue were analyzed using the extraction protocol previously described [32], with a detailed 32-step-protocol explained in the latter. The goal of this lysis process is to fractionate proteins based on their cellular compartmentalization. The sequential separation allows the recovery of a predicted protein in its compartment of 75–90% as previously described [30, 32]. Briefly, dissected frozen hemi-forebrain tissues (125–200 mg) are gently dissociated in NP40-lysis buffer (50 mM Tris–HCl [pH 7.6], 0.01% NP-40, 150 mM NaCl, 2 mM EDTA, 0.1% SDS) and centrifuged at 800×*g*, to separate extracellular proteins contained in the supernatant. The remaining loose pellet is then lysed with TNT-lysis buffer (50 mM Tris–HCl, pH 7.4, 150 mM NaCl, 0.1% Triton X-100), and centrifuged at 16,100×*g*, to separate intracellular proteins present in the aqueous phase. The subsequent pellet is finally dissociated in RIPA-lysis buffer (50 mM Tris–HCl, pH 7.4, 150 mM NaCl, 0.5% Triton X-100, 1 mM EDTA, 3% SDS, 1% deoxycholate) and centrifuged at 16,100×*g*, to separate membrane-bound proteins present in the supernatant. All supernatants were ultra-centrifuged for 20 min at 100,000×*g*. Before analysis, fractions were depleted of endogenous immunoglobulins by incubating lysates with 50 µL of Protein A-Sepharose, Fast Flow^®^ beads for one hour at 4 °C, followed by 50 µL of Protein G-Sepharose, Fast Flow^®^ beads (GE Healthcare Life Sciences). Protein amounts were determined with the Bicinchoninic acid protein assay (BCA Protein Assay, Pierce™).

### Antibodies

The following primary antibodies were used in this study: 6E10 [1:2,000] (Catalog no. SIG-803003, BioLegend), 4D6 anti-α-Synuclein [1:500–10,000] (Catalog no. SIG-39725, BioLegend), anti-β-Synuclein [1:5000] (Catalog no. ab76111, Abcam), anti-ASO (gift from Dr. Tracy Cole at Ionis Pharmaceuticals Inc.) [1:10,000], anti-NeuN [1:500] (Catalog no. ABU78, EMD Millipore), anti-Egr1 [1:100] (Catalog no. 22008-1-AP, Proteintech). The following secondary antibodies were used in this study: Alexa Fluor™ (Molecular Probes, Invitrogen) Goat-anti-Chicken 488 (Catalog no. A-11039), 568 (Catalog no. A-11041), 647 (Catalog no. A-21449), Goat-anti-Mouse 568 (Catalog no. A-11004), 647 (Catalog no. A-21235), Goat-anti-Rabbit 488 (Catalog no. A-11034), 555 (Catalog no. A-21435), 568 (Catalog no. A-11036), DyLight^®^ Goat-anti-Mouse 405 (Catalog no. 35501BID), IRDye^®^ (Li-COR) 800cw Goat anti-Rabbit (Catalog no. 925–32,211), IRDye^®^ (Li-COR) 680LT Goat anti-Mouse (Catalog no. 925-68020).

### Egr1 immunofluorochemistry and spot analysis

30 µm mouse brain sections were prepared using a vibratome (Leica) and stained with anti-Egr1 (Proteintech, Catalog no. 22008-1-AP, [1:100]). Briefly, freely floating sections were permeabilized with 0.3% Triton-X in PBS for 2 h at room temperature, and blocked with 0.3% Triton-X in PBS containing 10% donor goat serum for one hour before incubation at 4° C with primary antibodies in blocking solution. Detection was performed using Alexa Fluor™ conjugated secondary antibodies (Invitrogen). Slices were treated for autofluorescence with 1% Sudan Black solution and cover slipped with ProLong™ Diamond Antifade Mountant (Invitrogen, Catalog no. P36961). Digital images were obtained using a Leica Stellaris 8 confocal microscope. Raw image z-stacks of two slices per animal were analyzed by a blinded investigator using Imaris software suite (version 9.3, Bitplane Inc., USA). The spot analysis function was utilized to generate cell counts. FIJI software [51] was used to calculate hippocampal subfield areas for density measurements.

### Barnes circular maze

The apparatus used was an elevated circular platform (0.91 m in diameter) with 20 holes (5 cm diameter) around the perimeter of the platform, one of which was connected to a dark escape recessed chamber (target box) (San Diego Instruments, USA). The maze was positioned in a room with large, simple visual cues attached on the surrounding walls. The protocol used here was published elsewhere (http://www.nature.com/protocolexchange/protocols/349). Briefly, mice were habituated to the training room prior to each training day for 30 min in their cages. In addition, on the first day mice were placed at the center of the maze in a bottomless opaque cylinder for 60 s to familiarize the animals with the handling. Training sessions started 15 min later. Acquisition consisted of 4 trials per day for 4 days separated by a 15-min inter-trial interval. Each mouse was positioned in the center of the maze in an opaque cylinder, which was gently lifted and removed to start the session. The mice were allowed 180 s to find the target box on the first trial; all trials were 3 min long. At the end of the first 3 min, if the mouse failed to find the recessed escape box, it was gently guided to the chamber and allowed to stay in the target box for 60 s. The location of the escape box (pink circles on track plots) was kept constant with respect to the visual cues, but the location of the target hole was changed randomly. An animal was considered to find the escape chamber when its head and shoulders were above the escape box. An animal was considered to enter the escape chamber when the animal’s entire body was in the escape chamber and no longer visible on the platform. Memory retention was tested 24 h after the last training session (Probe trial day 5). Briefly, the escape hole was blocked for the duration of the probe, and the time spent searching the target quadrant for the escape hole was measured for each mouse. The same parameters were collected during acquisition and retention phases using the ANY-maze software (Stoelting Co., USA).

### Spatial maze path analysis

For each day during the acquisition phase of the task, track plots were obtained for each trial after video analysis using the ANY-maze^®^ software (Stoelting Co., IL, USA). Traveled paths were then classified visually using a paradigm established by prior studies [45]. Briefly, run patterns were scored as follows: 1, thigmotaxis; 2, random search; 3, scanning; 4, chaining; 5, directed search; 6, focal search; and 7, direct path. Based on this scoring scheme, 1–3 reflected non-spatial hippocampal learning, while search strategies 4–7 were considered to represent spatial hippocampal learning.

### Measurements of amyloid plaques

#### Immunofluorescence

Immunolabeling was performed to stain amyloid plaques using 6E10 antibodies. Brain tissue was permeabilized with 0.1%TritonTM-X100 then incubated in 10% Normal Goat serum to prevent nonspecific binding. Afterwards, the tissue was incubated with primary antibodies for 1 h using the Biowave^®^ Pro system (Pelco), followed by a series of PBS washes (3 × 6 min), and with secondary antibodies for 1 h.

#### Confocal imaging

Triple-label immunofluorescence was performed as previously described in [29] using Alexa Fluor™ − 488, − 555, − 647–conjugated secondary antibodies (Molecular Probes, Invitrogen), treated for autofluorescence with 0.1% Sudan Black solution, and coverslipped with ProLongGold mounting medium (Molecular Probes). Digital images were obtained using an Olympus IX81 FluoView1000 microscope. Raw image z-stacks were analyzed using Imaris9.3 software suite (Bitplane Scientific Software).

### Western blotting

Protein concentrations were determined by using the Pierce™ BCA protein assay.

#### Electrophoresis

Protein separation was done using SDS-PAGE on freshly prepared 12% SDS–polyacrylamide gels, pre-cast 10–20% SDS–polyacrylamide Tris-Tricine gels, or 10.5–14% or 4–10.5% Tris–HCl gels (Bio-Rad). Protein levels were normalized by using 2–100 µg of protein per sample (depending on the targeted protein). The samples were resuspended with 4X Tricine loading buffer and boiled for 5 min prior to loading.

#### Western blotting

Proteins were transferred to 0.2 µm nitrocellulose membrane (Bio-Rad) following electrophoresis. For primary neuron experiments, membranes were blocked and antibodies were diluted into Odyssey Blocking Buffer (TBS version; LI-COR Biosciences, USA). For all other experiments, membranes were blocked in TBS containing 5% bovine serum albumin (BSA; Sigma) for 1–2 h at room temperature, and probed with the appropriate antisera/antibodies diluted in 5% BSA-TTBS (TBS with 0.1% Tween-20). Primary antibodies were probed with either anti-IgG immunoglobulins conjugated with biotin, HRP or IR dyes (LI-COR Biosciences). When biotin-conjugated secondary antibodies were used, HRP- or IR-conjugated Neutravidin^®^ (Pierce) or ExtrAvidin^®^ (Sigma) was added to amplify the signal. Blots were revealed on a LI-COR Odyssey imaging platform (Li-Cor Biosciences).

#### Stripping

For re-probing, membranes were stripped using Restore™ Plus Stripping buffer (Pierce) for 5–180 min at room temperature, depending on the antibody affinity.

#### Quantification

Densitometry analyses were performed using the LI-COR Odyssey software. Each protein of interest was probed in 3 individual experiments under the same conditions. Quantification by software analysis, expressed as DLUs, followed determination of experimental conditions ascertaining linearity in the detection of the signal. This method allows for a dynamic range of ~ 100-fold above background. Respective averages were then determined across the triplicate Western blots. Normalization was performed against actin, βIII-tubulin or NeuN, which were also measured in triplicate. The color of the signal detected at 680 nm (red by default on the Odyssey) was modified to magenta to allow colorblind individuals to distinguish both channels.

### Acute slice electrophysiology

We collected acute coronal hippocampal slices (350 μm thick) from 8-month-old αSyn-KO mice. Slices were kept in ice-cold artificial CSF (ACSF) containing the following (in mM): 124 NaCl, 5 KCl, 1.25 NaH_2_PO_4_, 2 MgSO_4_, 26 NaHCO_3_, 2 CaCl_2_, and 10 glucose, gassed with 95% O_2_/5% CO_2_, pH 7.3–7.4. Slices were incubated in ACSF at room temperature for at least 1 h before use and then transferred to an immersion recording chamber, superfused at 2 mL/min with gassed ACSF, and visualized under a Nikon Eclipse E600FN microscope (Tokyo, Japan). Picrotoxin (50 μm) and CGP54626 (1 μm) were added to the solution to block the GABA_A_ and GABA_B_ receptors, respectively. Field excitatory postsynaptic potentials (fEPSPs) were evoked in the CA1 stratum radiatum by stimulating Schaffer collaterals (SCs) with tungsten microelectrodes (World Precision Instruments, Sarasota, FL) and recorded with ACSF-filled glass pipettes (< 5 MΩ). Recordings were obtained with PC-ONE amplifiers (Dagan Instruments). Signals were filtered at 1 kHz and acquired at 10 kHz sampling rate and fed to a computer through a DigiData 1322A interface board. pCLAMP version 10.4 (Axon Instruments) software was used for stimulus generation, data display, acquisition, and storage. For baseline recording, the stimulation intensity was adjusted to obtain 40% of the maximum slope of the response and inputs were stimulated (1 ms pulse duration) every 3 s. The slope of the fEPSPs was measured between 30 and 70% of maximum. For LTP induction, a tetanic stimulation (4 trains at 100 Hz for 1 s; 30 s intervals) was applied in the SC. fEPSP slope was normalized to 10 min of baseline recording. After LTP induction, fEPSPs were recorded for 60 min. The presence of LTP was determined by comparing the last 5 min of baseline with the last 1 min of recording. Groups were compared using a one-way ANOVA. When data did not meet normality, a one-way Kruskal–Wallis test with Dunn's method post hoc was applied.

### NanoString^®^ nCounter^®^ profiling

The nCounter^®^ mouse Neuropathology panel was purchased from NanoString (NanoString Technologies, USA) and processed using the nCounter Flex analysis system (NanoString Technologies, USA). Using nSolver 4.0 software, data underwent quality control for assay efficiency, background subtraction using the mean of negative controls, and normalization using positive controls and housekeeping genes (also called CodeSet content normalization) according to manufacturer instructions. Analysis of differentially expressed genes was conducted by one or two-way ANOVA using JMP Genomics version 9.0 (SAS Institute, USA) and a false discovery rate adjusted significance threshold of 0.05. One sample was determined to be an outlier and was removed from subsequent analysis.

### Pathway analysis using g:Profiler

The list of differentially regulated genes obtained through NanoString^®^ nCounter^®^ analysis was subjected to g:Profiler. The functional enrichment analysis was performed using g:Profiler (version e105_eg52_p16_e84549f) with g:SCS multiple testing correction method applying significance threshold of 0.05 (Raudvere, Kolberg et al., 2019). The following data sources were used: GO Molecular Function (GO:MF), GO Cellular Component (GO:CC), GO Biological Process, KEGG, Reactome, WikiPathways, TRANSFAC, miRTarBase and CORUM.

### STRING annotation & network visualization with Cytoscape

The list of differentially regulated genes obtained through NanoString^®^ nCounter^®^ analysis was subjected to STRING v11.5 via the stringApp v1.7.0 for Cytoscape v3.9.1 to identify and visualize possible gene clusters (Shannon et al., 2003; Szklarczyk et al., 2017). Data source settings for nodal associations were set to default with a confidence cutoff of ≥ 0.4 interaction score minimum. Gene networks were visualized using Cytoscape v3.9.1 software. Each node represents a protein, while each edge corresponds to coexpression subscores. Unless indicated otherwise, the node color reflects whether the gene is up- (orange) or downregulated (blue) while the color gradient reflects the magnitude of the change (the darker the more changed). The thickness of each edge is proportional to the STRING-based coexpression interaction between two genes (the thicker the edge, the stronger is their STRING-based association).

### Statistical analyses

When variables were non-normally distributed, nonparametric statistics were used (Spearman rho correlation coefficients, Kruskal–Wallis non-parametric analysis of variance followed by Bonferroni-corrected two-group *posthoc* Mann–Whitney U tests). When variables were normally distributed, the following parametric statistics were used (one/two/three-way ANOVA or RM-ANOVA followed by Bonferroni-corrected two-group *posthoc* Student t tests or Tukey HSD test). Sample size was determined by power analysis to be able to detect statistically significant changes within a 20% variation of measured responses. Analyses were performed using JMP Pro and JMP Genomics (SAS Institute, USA). Statistical tests and outcomes are compiled in, Additional File 2: Table S2.

## Results

### ASO-mediated reduction of αSyn mRNA and protein in wild-type mice

For gapmer ASOs to be a viable therapeutic tool, they must selectively reduce their intended messenger RNA and be well tolerated. We tested two different ASO constructs, named ASO1 and ASO2, targeting *SNCA* transcripts using in vitro and in vivo paradigms (Additional file 1: Fig. S1). First, we exposed primary mouse cortical neurons to acute applications of these two different ASOs (300 µg bolus) and measured the relative expression of *SNCA* mRNAs over time (Additional file 1: Fig. S1A). As expected, *SNCA* mRNA transcripts decreased over time with either ASO1 or ASO2 compared to PBS controls. ASO2 was less effective in lowering *SNCA* mRNA abundance, with only the 48-h time point showing a decrease. By contrast, ASO1 was more effective, with a ~ 60% decrease in expression compared to the ~ 30% decrease caused by ASO2.

**Fig. 1:**
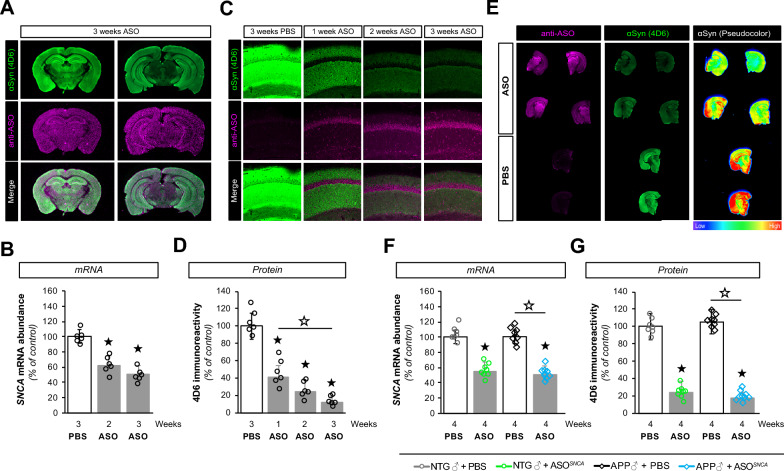
ASO1 disperses throughout the brain and lowers *SNCA* gene expression. **A** Infra-red imaging documenting the widespread distribution of ASOs 3 weeks after injection using anti- ASO (pink) and anti-αSyn (4D6, green) antibodies. **B** Reduction in *SNCA* mRNA at 2 and 3 weeks post-injection as determined by RT-qPCR. **C** Confocal imaging illustrating the presence of ASO1 (pink) and a corresponding decrease in αSyn (green) in mouse hippocampi. **D** Quantification of hippocampal 4D6 immunoreactivity in ASO1 or PBS treated mice. **E** Infra-red imaging detected αSyn (green) and ASO (pink) in coronal brain sections from PBS and ASO treated mice. The relative αSyn signal was lower in ASO-injected animals than in PBS-injected animals (pseudocolor). **F**, **G** Measurements of *SNCA* mRNA abundance by RT-qPCR (**F**) and αSyn protein amounts by immunofluorescence (**G**) in transgenic (APP) and non-transgenic (NTG) animals treated with PBS or ASO. Histogram bars represent mean ± SEM, ★P < 0.05 compared to NTG + PBS, ☆P < 0.05 compared to APP + PBS

Since ASO1 demonstrated a strong inhibition of SNCA mRNA expression in cultured mouse neurons, we tested ASO1 in mice. Because we demonstrated that a 300 µg bolus of ASO1 was sufficient to lower *SNCA* transcripts in vitro, mice were unilaterally injected with the same 300 µg dose of ASO1 into the lateral ventricle. To document that ASO1 was equally distributed across cerebral hemispheres following ICV delivery, we performed immunohistochemical labeling and whole-section imaging (Fig. 1A). Similarly to previous reports [17], we observed that ASO1 was homogeneously localized throughout the brain 3 weeks following injection using infrared-based imaging (Fig. 1A, Additional file 1: Fig. S1B). αSyn was detected with the 4D6 antibody and used as internal control.

To assess when ASO1 was most effective in knocking down *SNCA* mRNAs, we measured *SNCA* transcriptional gene products at 2 and 3 weeks post-ASO delivery by RT-qPCR. We found that the relative abundance of *SNCA* transcripts was reduced by 34% and 48% respectively (Fig. 1B). To determine the consequence of the ASO1-induced knockdown on αSyn protein levels in targeted areas (*e.g.*, hippocampus), confocal image analyses revealed a time-dependent reduction in αSyn and confirmed the presence of ASO1 in hippocampal cells, mirroring the observations made using infrared-based imaging (Fig. 1C). Specifically, the amount of hippocampal αSyn decreased by ~ 60, ~ 70 and ~ 80% at 1, 2 or 3 weeks post-ASO injection respectively (Fig. 1D). These results indicated that ASO1 suppressed αSyn mRNA and protein expression for several weeks following ICV injection.

To ensure that the ASO1 injections did not elicit an overt inflammatory reaction, we assessed the immunoreactivity profiles of ionized calcium binding adaptor molecule 1 (Iba1) and glial fibrillary acidic protein (GFAP), markers of microgliosis and astrocytosis respectively in saline and ASO1-injected mice (Additional file 1: Fig. S1C). There were no differences in Iba1 or GFAP immunoreactivity between groups (Additional file 1: Fig. S1D,E).

Overall, these findings obtained in mice demonstrated that ASO1 reduced *SNCA* gene transcription in mouse neurons and brain tissues in absence of overt side effects.

### Partial inhibition of ɑSyn expression in a mouse model of Alzheimer’s disease

We previously reported that constitutive genetic deletion of *SNCA* in the J20 transgenic mouse model of AD rescues premature mortality, memory and synaptic deficits despite an exacerbation of amyloid pathology [25]. To test whether a transient lowering of αSyn could be similarly beneficial, we performed a translational study in 6-month-old non-transgenic (NTG) and J20 APP transgenic (APP) mice to assess the therapeutic potential of ASO1 on cognitive function. With this new cohort of animals, we opted to extend the intervention window to 4 weeks post-ICV delivery to minimize potential undesired effects caused by inflammatory reactions to the procedure. We first examined the distribution of ASO1 throughout the brain and measured the relative expression of *SNCA* mRNA (Fig. 1E, F). ASO1 was readily detected by antibodies raised against the chemical backbone of the ASO in animals treated with ASO1, while brain sections from PBS-injected animals showed no apparent signal (Fig. 1E, *left panel*). Consistent with our previous results, the new cohort of NTG animals injected with ASO1 also displayed a ~ 50% decrease in *SNCA* mRNA abundance (Fig. 1F). The amount of *SNCA* mRNA present in saline-treated APP transgenic animals was indistinguishable from that measured in control NTG littermates. More importantly, *SNCA* mRNA expression was reduced by half following ASO1 delivery in APP transgenic mice compared to APP animals injected with PBS. In addition, the lowering in *SNCA* transcripts was identical across ASO-treated groups, demonstrating the consistency of the knockdown induced.

We next quantified the amount of αSyn protein expression (Fig. 1E, G, Additional file 1: Fig. S2). The effect of ASO1 on αSyn brain abundance was further supported by pseudocolor images of αSyn (4D6), which readily showed higher amounts of αSyn protein in PBS-injected mice compared to ASO1-injected mice (Fig. 1E*, right panel*). This qualitative difference was further supported by quantitative analyses documenting that ASO1 ICV injection reduced hippocampal αSyn protein by ~ 80% in both APP and NTG animals (Fig. 1G). Lastly, αSyn protein abundance was measured by western blotting, which confirmed a ~ 50–60% decrease in the forebrain of ASO1-injected mice compared to saline-injected animals (Additional file 1: Fig. S2). These findings indicate that ɑSyn-targeting ASOs consistently and robustly lower *SNCA* mRNA and ɑSyn protein in vivo.

### Lowering ɑSyn rescues cognitive deficits in male APP mice

**Fig. 2:**
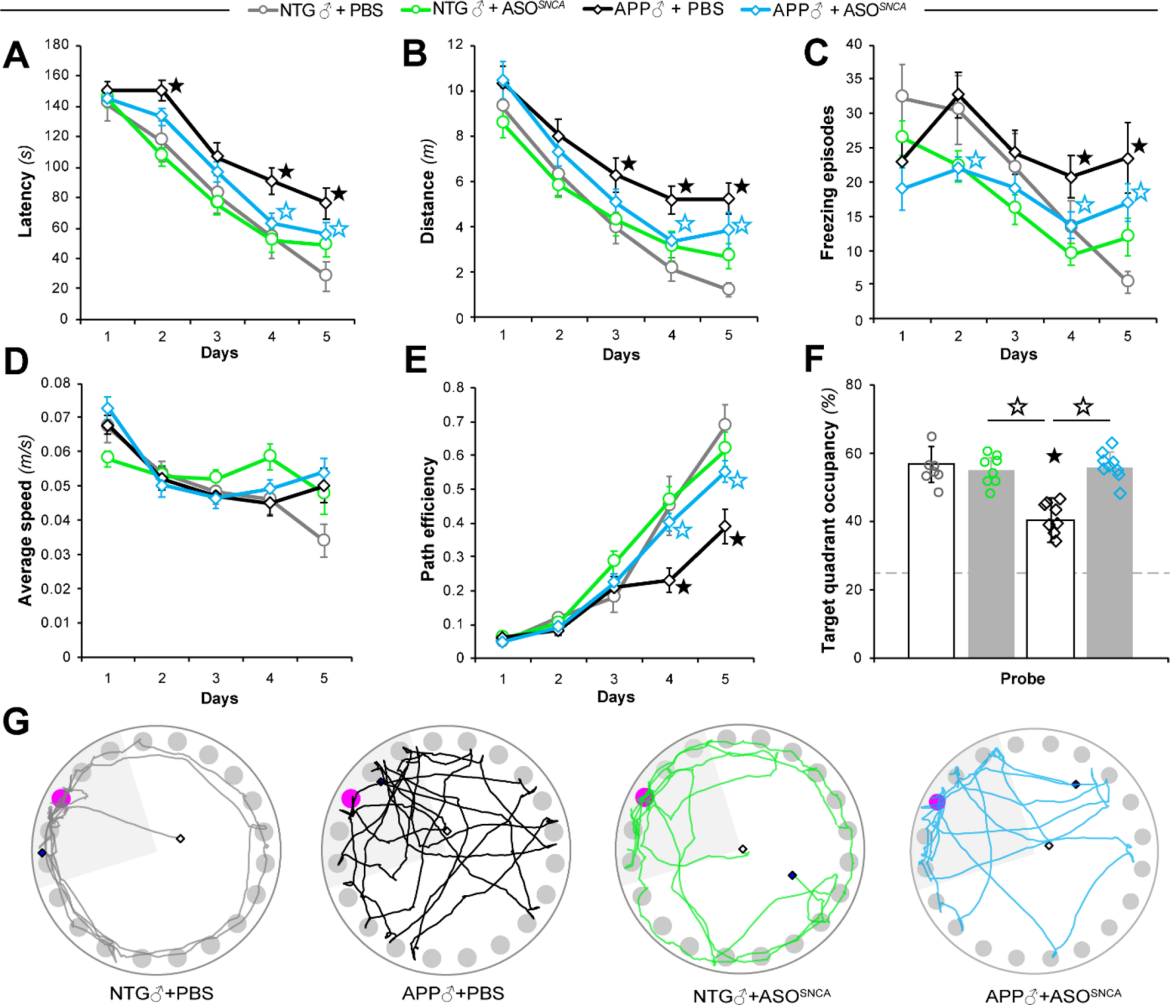
Behavioral effects of αSyn reduction in male NTG and APP mice. Male APP and NTG controls were tested in the Barnes circular maze (BCM) at six months of age. **A**, **B** Spatial learning of the BCM task was reflected by reductions in escape latency (**A**) and distance traveled (**B**). **C**, **D** Measurements of freezing episode numbers (**C**) and average animal speed (**D**) in the BCM task were used to assess phenotypic changes other than spatial learning. **E** Quantitative assessment of animal trajectories during the learning phase using path efficiency. **F**, **G** Spatial memory retention in the BCM task was determined by target quadrant occupancy (**F**) and by assessment of path traces (**G**). Data represent mean ± SEM; ★*P* < 0.05 compared to NTG + PBS, ☆*P* < 0.05 compared to mice of the same genotype in the other treatment group, *n* = 8 mice/treatment/genotype

**Fig. 3:**
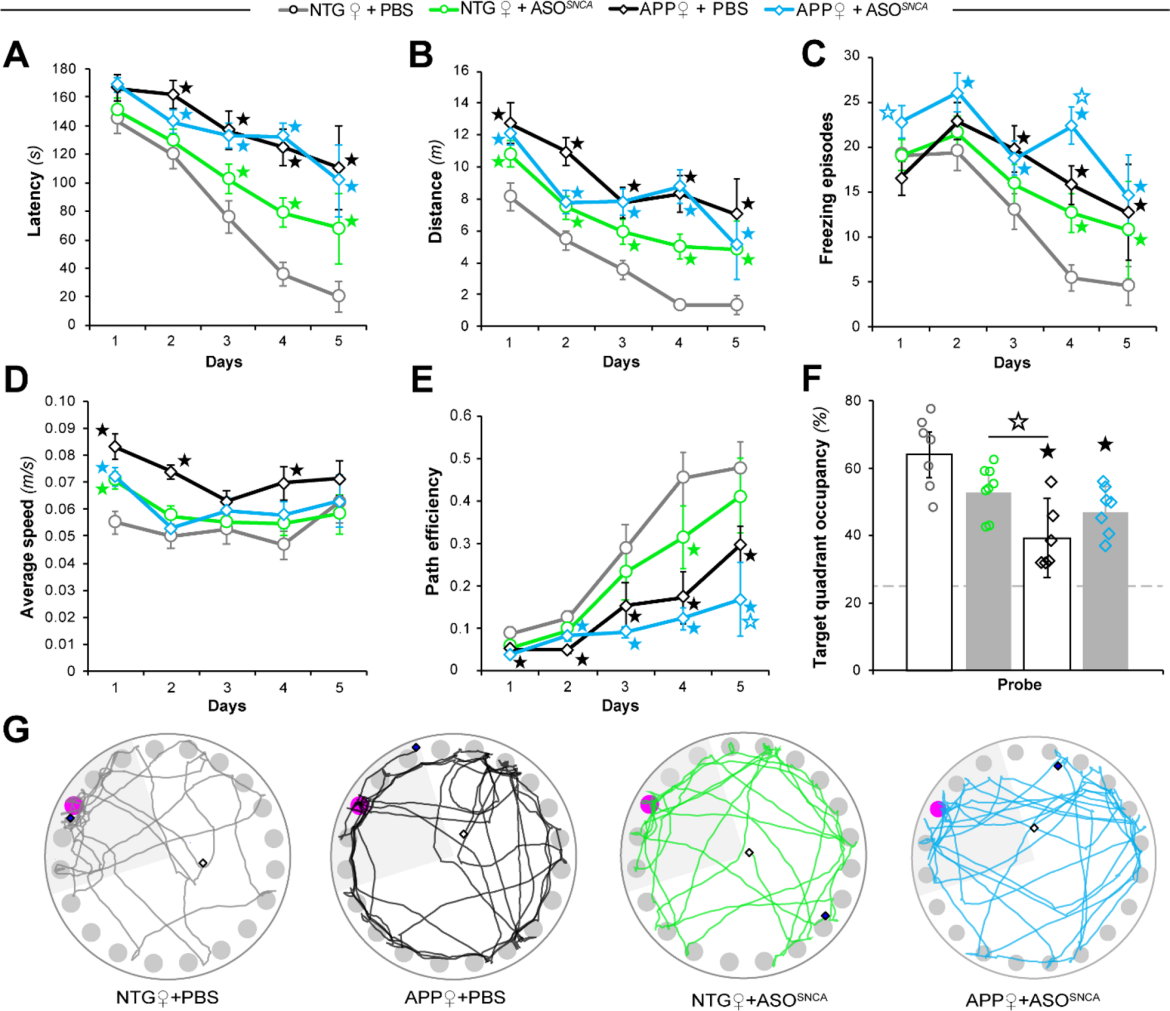
Behavioral effects of αSyn reduction in female NTG and APP mice. Female APP and NTG controls were tested in the Barnes circular maze (BCM) at six months of age. **A**, **B** Spatial learning of the BCM task was reflected by reductions in escape latency (**A**) and distance traveled (**B**). **C**, **D** Measurements of freezing episode numbers (**C**) and average animal speed (**D**) in the BCM task were used to assess phenotypic changes other than spatial learning. (**E**) Quantitative assessment of trajectories used by animals during the learning phase using path efficiency. **F**, **G** Spatial memory retention in the BCM task was determined by target quadrant occupancy (**F**) and by assessment of path traces (**G**). Data represent mean ± SEM; ★*P* < 0.05 compared to NTG + PBS, ☆*P* < 0.05 compared to mice of the same genotype in the other treatment group, *n* = 8 mice/treatment/genotype

We previously demonstrated that ablating the *SNCA* gene in APP mice improved cognition using a genetic approach [25], suggesting that reducing ɑSyn expression might constitute a putative translational strategy to alleviate memory deficits. To examine this possibility, both APP and NTG mice were subjected to the Barnes circular maze (BCM), a hippocampal-dependent spatial reference memory task, four weeks post-injection. In contrast to our earlier genetic study which only used male mice [25], and given the importance of sex as a biological variable for biomedical research, here we evaluated both male (Fig. 2) and female animals (Fig. 3).

During the acquisition phase of the task, the male PBS-injected NTG and APP mice (NTG + PBS and APP + PBS respectively) were able to learn the task as assessed by decreased latencies. However, APP animals displayed a mild acquisition deficit (Fig. 2A), consistent with previous reports [25, 30]. APP mice that received a single bolus of ASO1 (APP + ASO^*SNCA*^) displayed decreased latency compared to saline-injected APP littermates. There was no difference in the latencies of the ASO-injected APP and NTG mice on day 5 (Fig. 2A). Paralleling these observations, similar changes were also observed for the distance traveled by the animals during the task (Fig. 2B), suggestive of better spatial memory function.

However, APP mice are well-known to be hyperactive [25, 62] and we previously documented that *SNCA* deletion can alleviate this hyperactivity phenotype [25]. As expected, saline-injected APP mice traveled the most during the 5-day acquisition stage. Importantly, the ASO-injected APP mice traveled shorter distances to the escape hole than the PSB-injected APP mice (Fig. 2B). There was no difference in distance traveled between the ASO-injected APP and the NTG mice on any acquisition learning day (Fig. 2B), likely the result of an amelioration of hyperactive behavior.

Genetic deletion of *SNCA* also normalized the characteristic freezing behavior of APP mice to baseline levels of NTG littermates [25]. Consistent with our previous study, APP + PBS mice froze more than NTG + PBS or NTG + ASO^*SNCA*^ groups (Fig. 2C). The APP + PBS mice also froze more than ASO-injected APP mice, whose behavior was indistinguishable from the NTG groups. This alleviated freezing behavior in APP + ASO^*SNCA*^ animals indicated that a reduction in *SNCA* transcriptional products can return APP mice to a normal behavioral phenotype.

Of note, there were no differences in average speed between any of the groups (Fig. 2D). These findings likely indicated that the decrease observed in distance traveled was not simply due to a global reduction in locomotor behavior in APP mice but perhaps corresponded to more efficient trajectories during the learning phase.

To directly address this possibility, path length efficiency, defined as the path taken by the mouse compared to the shortest possible route to the escape hole, was measured. By the end of the acquisition phase, all groups but the APP + PBS mice displayed robust increases in path efficiency (Fig. 2E). Importantly, the ASO-injected APP mice were more efficient than the PBS-injected APP mice on days 4 and 5. Altogether, measures of latency, distance traveled and path length efficiency were consistent with better spatial learning memory in APP + ASO^*SNCA*^ mice compared to PBS-treated APP animals.

On the final day of the BCM task, a probe trial was conducted to evaluate memory retention in all tested groups. As expected, the target quadrant occupancy for the NTG + PBS group was higher than that of the APP + PBS group (Fig. 2F). Injecting *SNCA*-targeting ASO1 in NTG mice did not alter retention compared to NTG + PBS animals. However, the APP + ASO^*SNCA*^ group performed significantly better than the APP + PBS group and similarly to both NTG groups (Fig. 2F). This apparent rescue in spatial memory retention was also reflected in the track plots from each group (Fig. 2G). These results indicate that both acquisition and retention of spatial reference memory was improved in male APP mice just four weeks after a single injection of ASO^*SNCA*^.

### Reducing ɑSyn selectively worsens cognition in female mice

In an effort to rigorously consider biological variables essential to neurological diseases, female APP mice were subjected to the same behavioral testing as the male mice (Fig. 3). As expected, NTG saline-injected female mice had shorter latencies to the escape hole than APP + PBS female mice (Fig. 3A). Unlike male animals, however, it was surprising that NTG + ASO^*SNCA*^ female mice had longer latencies than NTG + PBS mice on days 3–5. Importantly, APP mice that received ASO^*SNCA*^ displayed similar latencies to the APP + PBS female control mice, indicating that there was no effect of ASO administration on the female APP mice (Fig. 3A). This pattern repeated itself in the analysis of distance traveled, whereby ASO^*SNCA*^ injection increased the distances traveled by NTG females and failed to alter the distance traveled by APP mice (Fig. 3B).

Because both latency and distance traveled in the BCM task can be impacted by alterations in freezing behavior and animal speed, we analyzed both of those variables for each group of female mice. Although the number of freezing episodes decreased over training in NTG + PBS female mice, their number remained elevated in the NTG + ASO^*SNCA*^ group (Fig. 3C). Here again, ASO^*SNCA*^ injection did not decrease freezing behavior in APP mice, and in fact exacerbated it on days 1 and 4 (Fig. 3C). Next, we examined the average speed of the animals, an important variable when considering locomotor hyperactivity previously described in male J20 mice [62]. Apart from the first day, there was no difference in speed between the NTG + PBS females and the NTG + ASO^*SNCA*^ females (Fig. 3D). Unexpectedly, the APP + PBS females had higher average speeds than the NTG + PBS females on days 1, 2 and 4. By contrast, the average speeds displayed by APP + ASO^*SNCA*^ mice were similar to those of the NTG groups (Fig. 3D). However, unlike in males, reducing *SNCA* mRNA and protein did not rescue the hyperactivity and freezing phenotypes observed in APP animals, suggesting sex differences in αSyn expression or function.

As additional evidence of spatial learning, we evaluated path length efficiency. NTG females, whether injected with PBS or ASO, improved their path efficiency over time (Fig. 3E). APP + PBS females also improved their path efficiency, though not as much as NTG + PBS animals. The APP + ASO^*SNCA*^ females used less efficient paths to reach the escape target than the NTG + PBS and were largely like the APP + PBS female groups (Fig. 3E). Contrary to the effect we observed in male animals, administering ASO^*SNCA*^ worsened the spatial learning of female mice irrespective of transgene expression.

Next, we determined spatial reference memory retention by subjecting animals to a probe trial on the last day of the task (Fig. 3F). Even though target quadrant occupancy trended towards a decrease in NTG + ASO^*SNCA*^ animals compared to NTG + PBS mice, both groups were statistically similar. As expected, the APP + PBS females spent less time in the correct quadrant than both the NTG + PBS and the NTG + ASO^*SNCA*^ females (Fig. 3F). The NTG + ASO^*SNCA*^ females performed better than the APP + PBS group. Consistent with persisting learning deficits, ASO^*SNCA*^ injection did not improve the probe trial performance of the APP females, who performed no differently than the APP + PBS mice. The representative track plots from each group further supported the quantitative analysis of the probe trial and illustrated the lack of rescue in APP mice (Fig. 3G).

Despite well-established sex differences across multiple APP transgenic models whereby earlier-onset amyloid pathology has been consistently noted in females [22], gender effects on Aβ pathology have not been systematically reported for the J20 APP mouse model. For these reasons, and because transiently lowering αSyn could affect amyloid burden, we aimed to determine whether ASO^*SNCA*^ delivery altered Aβ deposition in J20 mice in a sex-specific manner. To this end, we measured amyloid burden using confocal imaging analysis (Additional file 1: Fig. S3A) as previously reported [25]. Quantitative analyses of hippocampal Aβ deposits revealed no differences in amyloid loads between sexes in 6-month-old APP + PBS or APP + ASO groups (Supplementary Fig. 3B, Additional file 1), arguing against a potential impact of Aβ pathology on the observed behavioral phenotype.

Altogether, these data revealed an unexpected sex difference in the response to αSyn-lowering ASOs in APP transgenic mice. Importantly, these findings also demonstrated gender-specific responses to mouse ASO^*SNCA*^ in NTG mice, whereby knock-down of *SNCA* transcripts resulted in learning and retention deficits of spatial memory in female mice compared to male mice.

### Differential role of αSyn on cognition in male and female mice

**Fig. 4:**
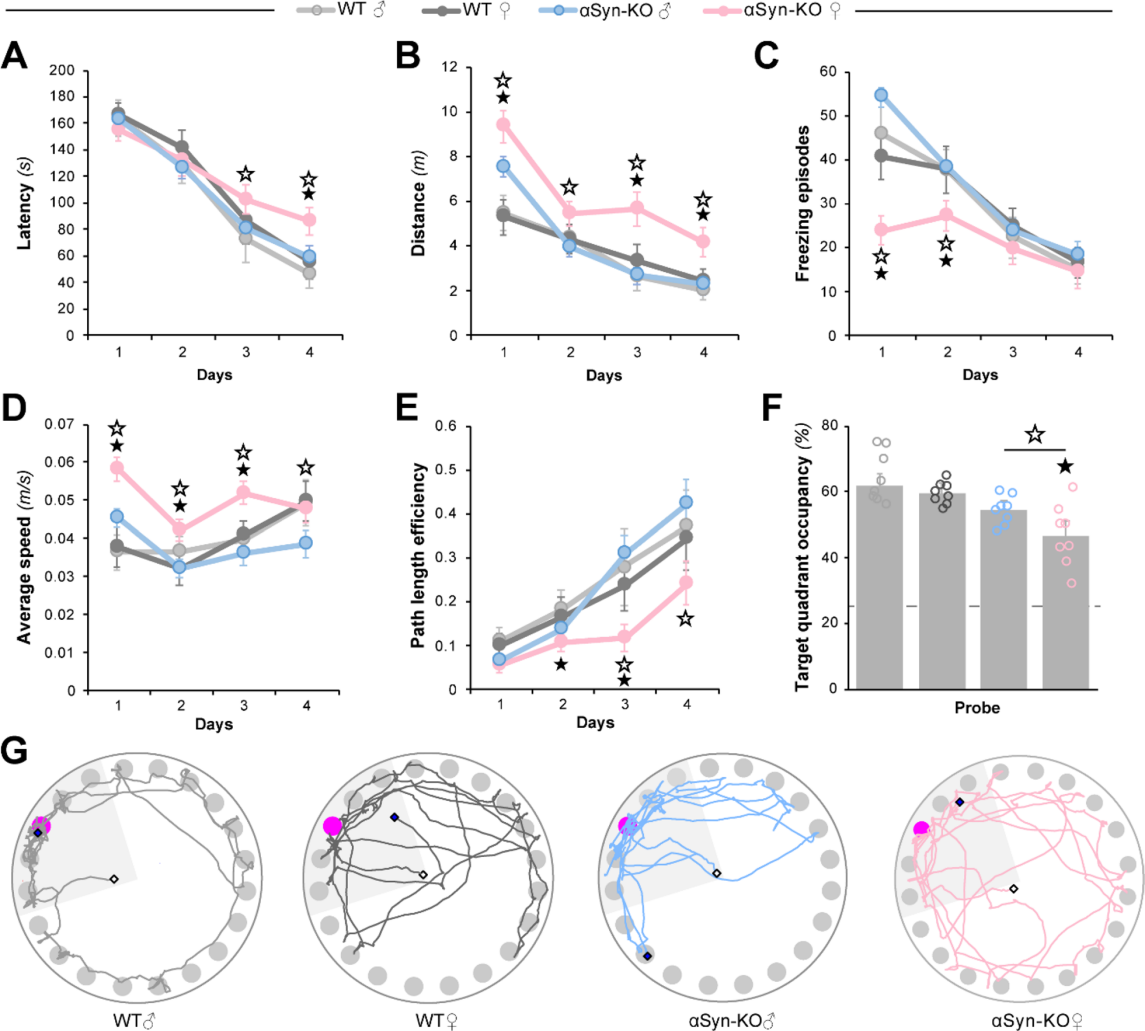
Genetic ablation of αSyn negatively impacts the performance of female mice on the Barnes circular maze. Male and female αSyn knock-out mice (αSyn-KO) and wild-type C57BL/6 J controls were tested in the Barnes circular maze (BCM) at six months of age. **A**, **B** Spatial learning of the BCM task was reflected by reductions in escape latency (**A**) and distance traveled (**B**). **C**, **D** Measurements of freezing episode numbers (**C**) and average animal speed (**D**) in the BCM task were used to assess phenotypic changes other than spatial learning. (**E**) Quantitative assessment of trajectories used by animals during the learning phase using path efficiency. (**F-G**) Spatial memory retention in the BCM task was determined by target quadrant occupancy (**F**) and by assessment of path traces (**G**). Data represent mean ± SEM; ★*P* < 0.05 compared to WT mice of the same sex, ☆*P* < 0.05 compared to mice of the same genotype and opposite sex, *n* = 8 mice/genotype/sex

Since the sex-specific behavioral effects observed as a result of the *SNCA* knockdown implied that αSyn might function differently in male and female brains, we investigated whether the genetic deletion of *SNCA* would differentially impact learning and memory in male and female animals. In line with previously published results from our group [25], αSyn-KO males performed no differently than age-matched non-littermate NTG mice based on latency (Fig. 4A) and distance (Fig. 4B) measurements. Female NTG mice were indistinguishable from the NTG and αSyn-KO males on both latency and distance parameters. To our surprise, and in sharp contrast with male animals, αSyn-KO female mice displayed clear deficits. They displayed longer latencies and traveled longer distances to reach the escape hole compared to both NTG females and αSyn-KO males, indicating that ablating αSyn was detrimental to the spatial learning of female mice (Fig. 4A, B; respectively).

To gain a more complete picture of other putative changes in αSyn-KO female mice, we also measured freezing and average speed to better characterize baseline locomotor activity in each group. Male and female NTG mice displayed equivalent freezing episodes (Fig. 4C) and had comparable average speeds (Fig. 4D). αSyn-KO males froze the same amount as NTG mice. At the beginning of the acquisition phase of the task, αSyn-KO females froze less often than both αSyn-KO male littermates and female NTG mice, though this behavior faded over time (Fig. 4C). The average speed of male and female NTG mice was equivalent, and αSyn-KO males were no different than the NTG groups. However, female αSyn-KO mice elicited greater average speeds than NTG females and male αSyn-KO littermates on days 1, 2 and 3. This difference also persisted on day 4 when compared to αSyn-KO males, though at that point of learning female αSyn-KO mice were no longer different from NTG animals (Fig. 4D). The combined observation that female αSyn-KO mice traveled larger distances, moved faster and with fewer freezing episodes during the first two days of training, was suggestive of a hyperactive phenotype early in the task.

Because this hyperactivity could also affect path length efficiency, we examined whether gender differences impacted spatial navigation. Male and female NTG mice took paths of equivalent efficiency to the escape hole, and αSyn-KO males were similarly efficient (Fig. 4E). Though all groups improved their path efficiency over time, female αSyn-KO mice were the least efficient and their spatial navigation was worse than that used by male αSyn-KO littermates on acquisition days 3 and 4 (Fig. 4E). To determine whether male and female αSyn-KO mice used similar learning strategies, track plot videos were analyzed as previously described [18, 45]. The relative distribution of different search strategies used by male and female αSyn-KO mice revealed that both groups relied on similar spatial learning strategies (Supplementary Fig. 4A, B, Additional file 1), possibly indicating a differential encoding of memory traces across sexes in αSyn-KO animals.

On the last day of testing, a probe trial was conducted to assess spatial memory retention. The NTG mice all demonstrated strong spatial memory retention, as they spent ~ 60% of their time in the target quadrant during that retention test (Fig. 4F). Male αSyn-KO mice spent the same amount of time in the target quadrant as the NTG mice. Female knockout mice, however, spent less time in the target quadrant than both female NTG mice and male αSyn-KO mice (Fig. 4F). The difference in target quadrant occupancy was also evident when comparing representative traces from each group (Fig. 4G).

Considering that potential sex-dependent differences in αSyn brain expression could exist, we measured the relative αSyn protein abundance in the hippocampi of WT mice while αSyn-KO mice served as internal negative controls (Supplementary Fig. 5, Additional file 1). No differences in hippocampal αSyn protein abundance were detected across sex. Because synuclein family members could compensate for the absence of αSyn, we also measured β-synuclein and found no differences across groups.

We next considered the possibility that female αSyn-KO mice could display an earlier onset of putative spatial memory deficits than αSyn-KO males. To directly test this hypothesis, we compared spatial learning and retention of young (6-month-old) and middle-aged (12-month-old) male αSyn-KO mice in the BCM task (Supplementary Fig. 6, Additional file 1). With the exception of a slight difference in average speed across age groups, all other variables examined (i.e. latency, distance traveled, freezing episodes and path efficiency) were indistinguishable (Additional file 1: Fig. S6A–E). Spatial memory retention was similar between young and middle-aged αSyn-KO males (Additional file 1: Fig. S6F), a conclusion further supported by corresponding track plots (Additional file 1: Fig. S6G).

With these potential interpretations excluded, these findings revealed that the function of αSyn in learning and memory differs between male and female brains.

### Differential role of αSyn on long-term potentiation in male and female mice

**Fig. 5:**
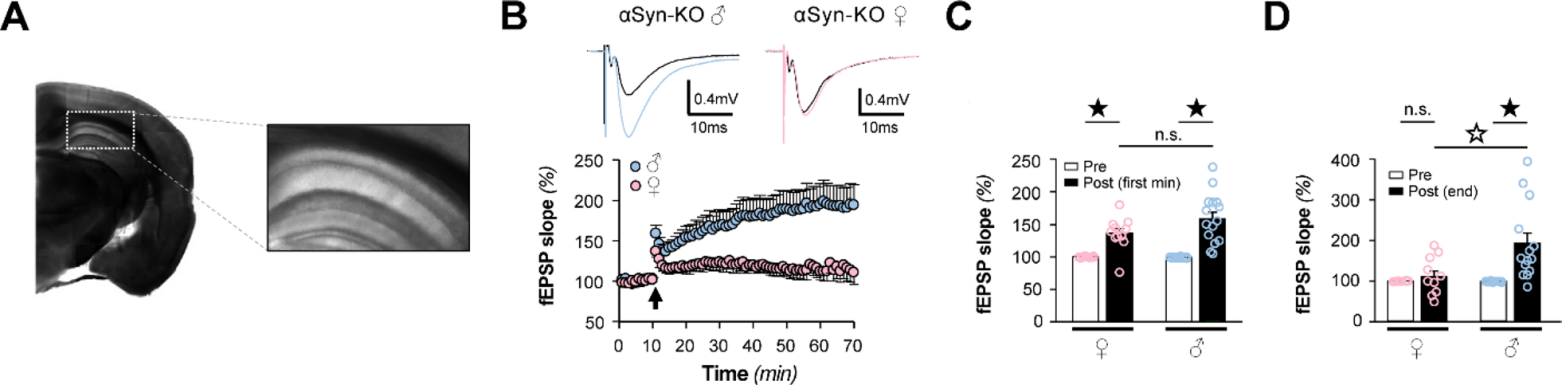
Sex differences in αSyn-KO mice hippocampal LTP. **A** Field excitatory postsynaptic potentials (fEPSPs) were recorded at CA1 synapses by stimulating the Schaffer collaterals. **B** *Top*, Representative fEPSP traces before (black) and after a high-frequency stimulation (HFS) of the Schaffer collaterals recorded from male (blue) and female (pink) αSyn-KO mice. *Bottom*, fEPSPs recorded from hippocampal CA1 during long-term potentiation induced by HFS of Schaffer collaterals (100 Hz, 1 s) of αSyn-KO females (n = 12, pink) and males (n = 14, blue). Arrowhead indicates application of HFS. **C**, **D** fEPSP slopes 1 min (**C**) and 60 min (**D**) following HFS (post), relative to baseline established prior to HFS (pre), from female and male αSyn-KO mice. Data are expressed as mean ± SEM; ★*P* < 0.05 compared to pre-HFS within the same sex group, ☆*P* < 0.05 compared to mice to opposite sex

We next determined whether long-term potentiation (LTP), a cellular mechanism widely assumed to underlie learning and memory, was altered in αSyn-KO mice based on sex. We induced LTP in acute hippocampal slices from 8-month-old αSyn-KO female and male mice (Fig. 5A). High-frequency stimulation (HFS) of the Schaffer collaterals elicited normal initial potentiation in both sexes (Fig. 5B, C). One minute following induction, the percent potentiation was 36.8 ± 6.9% in females and 58.8 ± 10.4% in male slices. However, this initial potentiation failed to stabilize in female slices, with responses decaying to control levels over the hour post HFS, while potentiation in male slices was stable and robust (Fig. 5B, D). Sixty minutes following HFS, the percent potentiation was 10.56 ± 14.6% in females and 94.14 ± 24.8% in males.

Together, these electrophysiological results demonstrate that LTP is impaired in female αSyn-KO hippocampal slices compared to male animals.

### Identification of a differentially expressed gene network centered around *EGR1* in the hippocampi of *SNCA*-null mice

**Fig. 6:**
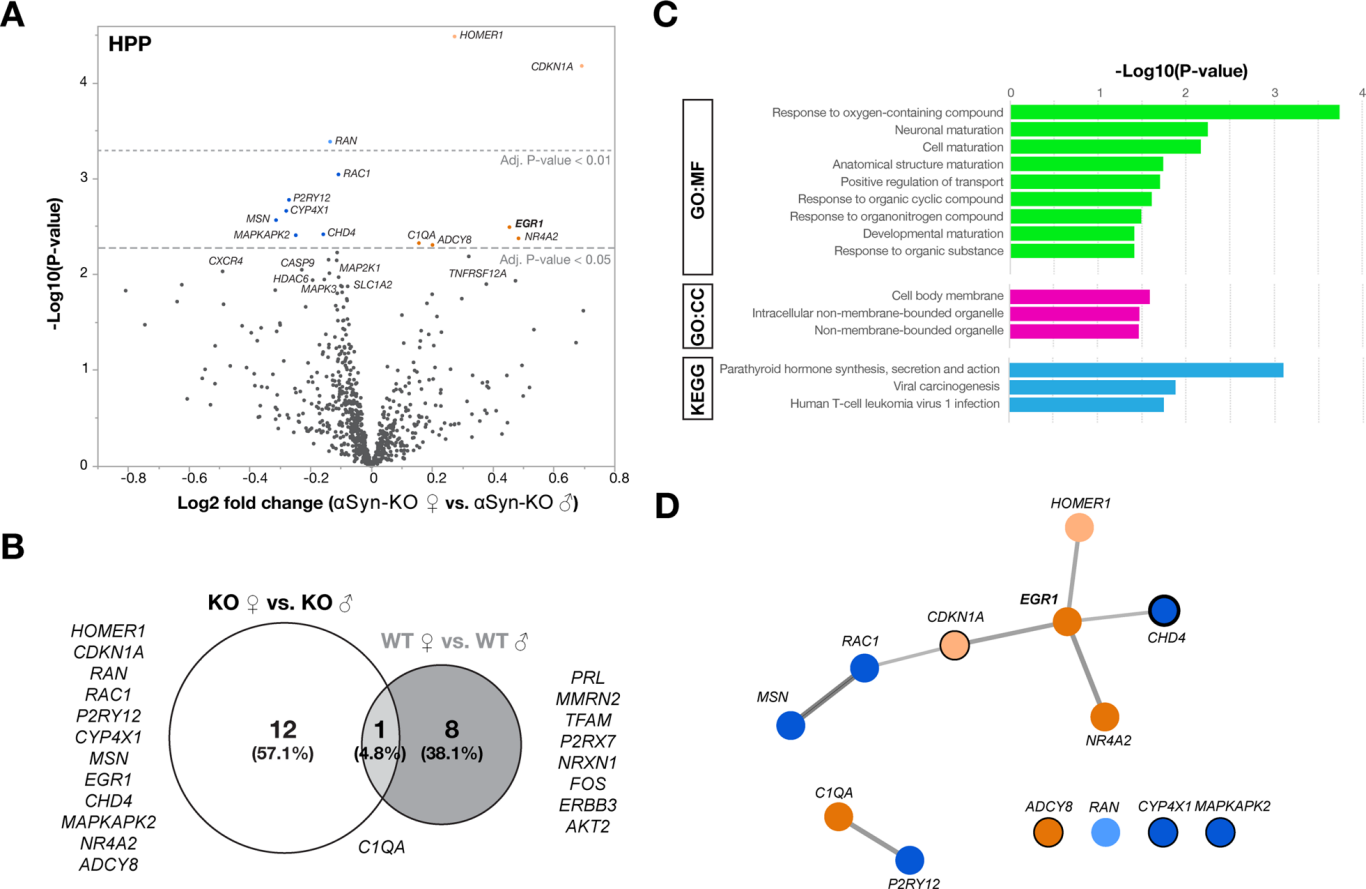
Differential gene expression in male and female αSyn-KO mice. NanoString^®^ transcriptional profiling was performed on hippocampal RNA from 6-month-old female and male αSyn-KO mice. **A** Volcano plot of the differentially expressed genes of αSyn-KO females compared to αSyn-KO males at adjusted significance thresholds of *P* < 0.05 and *P* < 0.01. Orange indicates upregulated genes, blue indicates downregulated genes. **B** Venn diagram of differentially expressed genes shows little overlap between WT and αSyn-KO females and males. **C** Top results for GO and KEGG databases using the female and male αSyn-KO differentially expressed gene list performed with g:Profiler for functional enrichment analysis. **D** MCL clustering and visualization of Cytoscape clusters was performed using clusterMaker2 based on co-expression data. Analysis revealed *EGR1* as a central node in the network as well as several *EGR1* target genes (black circles, *thickness reflects the number of responsive elements*)

To determine the underlying molecular mechanism supporting this sex-dependent role of αSyn on spatial learning and synaptic plasticity, we performed a NanoString^®^ transcriptional profiling of hippocampal tissues from wild-type (WT) and αSyn-KO mice based on sex. Unsupervised hierarchical clustering of the normalized dataset readily segregated hippocampal datasets by genotype, while sex representation was slightly more heterogeneous (Additional file 1: Fig. S7A). Principal component (PC) analysis revealed a dominant effect of PC1 (39%), resulting in the clustering of the animals based on genotype, whereas other PCs accounted for less than 8% (Additional file 1: Fig. S7B). Comparing female and male αSyn-KO mice, 13 genes were differentially expressed including *HOMER1A*, *CDK1NA*, *RAN*, *RAC1*, *P2RY12*, *CYP4X1*, *MSN*, *EGR1*, *CHD4*, *MAPKAPK2*, *NR4A2*, *C1QA* and *ADCY8* (Fig. 6A, B). Only one of these genes, *C1QA*, was also common with differentially expressed genes when comparing female and male hippocampi in WT mice (Fig. 6B). Pathway analysis identified response to oxygen-containing compound, neuronal maturation and cell maturation (Fig. 6C). Importantly, network analyses using Cytoscape [55] identified a differentially expressed gene network centered around *EGR1*, a central modulator of learning and memory [24, 60], in the hippocampi of αSyn-KO mice (Fig. 6D). Of note, 5 out of the 12 gene hits (*CDK1NA, CHD4*, *CYP4X1*, *MAPKAPK2* and *ADCY8*) correspond to gene targets of the transcription factor Egr1, also known as Zif268 (Fig. 6D, *black circles*).

**Fig. 7:**
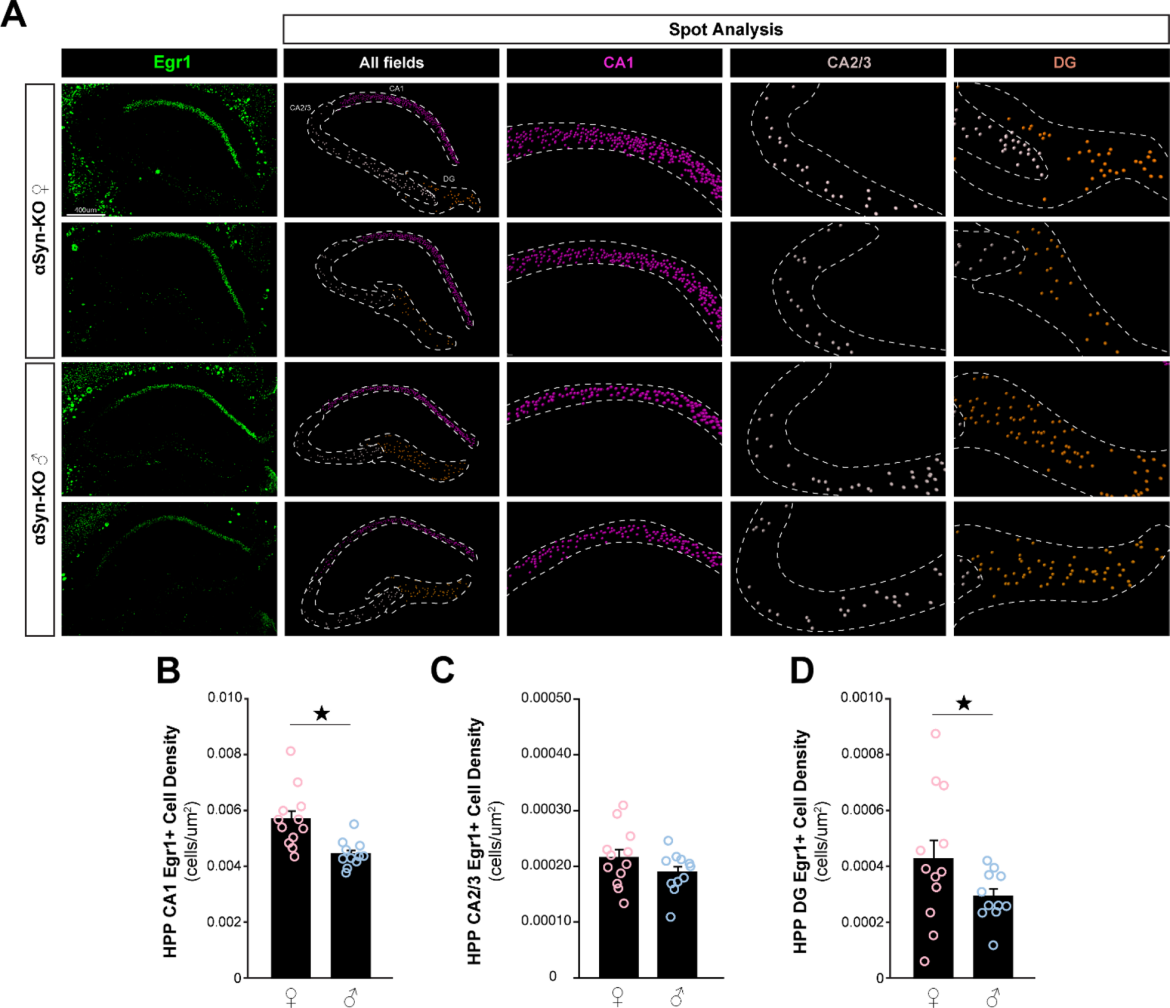
Egr1 protein expression in the hippocampus of αSyn-KO mice. Immunofluorescence labeling and confocal microscopy image analysis of hippocampal slices from male and female αSyn-KO mice were performed for Egr1 density. **A** Representative 20× confocal images for Egr1 (green) and corresponding segmentations using Imaris spot analysis (CA1, magenta; CA2/3, grey; DG; brown). Hippocampal regions are indicated by dashed white lines while Egr1-positive neurons are indicated by spots. **C**, **D** Cell density analysis of Egr1-expressing neurons in CA1 (**B**), CA2/3 (**C**) and dentate gyrus (DG; *D*). Histogram bars represent mean ± SEM, ★*P* < 0.05, n = 6 mice/sex, 2 slices/mouse

To validate this finding, we performed confocal image analyses following immunofluorescent labeling of Egr1 in male and female αSyn-KO mice (Fig. 7A). As previously documented [43], Egr1 was preferentially expressed in CA1 excitatory neurons and more sparely in other hippocampal fields, i.e. CA2, CA3 and dentate gyrus (DG). Using Imaris spot analysis, we found that Egr1-positive density was elevated in female mice compared to male littermates in the CA1 and DG (Fig. 7B, D). By contrast, no difference was observed in the CA2/3 region (Fig. 7C).

Overall, these results thus revealed a novel connection between αSyn and Egr1 in a sex-specific manner (Supplementary Fig. 8, Additional file 1).

## Discussion

Accumulating evidence from our group and others has implicated αSyn as a potentiator of AD [3, 13, 25, 29, 30, 56]. Based on the demonstration that *SNCA* ablation rescued cognitive deficits in male APP mice [25], we hypothesized that transient lowering of αSyn expression through the use of an ASO targeting *SNCA* mRNA would alleviate memory deficits in the same APP mouse model. We found that a single injection of a murine ASO^*SNCA*^ was effective at decreasing *SNCA* mRNA and protein abundance in mice. As predicted by our hypothesis, ASO^*SNCA*^ administration improved spatial reference memory in APP mice. Surprisingly, we observed this therapeutic benefit in male, but not female, animals. Furthermore, behavioral experiments using constitutive αSyn-KO mice revealed a sex-specific role of αSyn for spatial learning and memory. To gain functional insights into these differences across sexes, we performed electrophysiological recordings and transcriptional profiling. These experiments revealed previously unknown differences in hippocampal LTP between males and females, and in αSyn-dependent gene expression patterns, including a network of differentially regulated genes centered around *EGR1*, a critical modulator of long-term learning and memory. Overall, these results have two important consequences: 1) they provide a novel proof-of-principle demonstration that reducing αSyn expression in AD is a likely viable translational strategy only in male subjects, and 2) they document a novel function of αSyn on long-term spatial memory which differs between male and female brains.

### ASO-mediated knockdown of αSyn rescues memory deficits in male APP mice

ASOs have been increasingly used in recent years to modify protein expression in neurological disorders [5, 47]. Earlier studies in mouse models of spinal muscular atrophy (SMA) demonstrated that delivery of ASOs into the lateral ventricle were effective at modifying target mRNA splicing and functional protein abundance in brain tissue [41]. Human clinical trials yielded transformative results, with improved motor function and survival in children diagnosed with SMA who received the Nusinersen ASO (Spinraza^®^), which led to its rapid approval by the FDA [36]. The recent successes of ASO therapies in SMA and Duchenne muscular dystrophy [28] has sparked interest in applying similar strategies to other degenerative diseases [14, 47]. While synuclein proteins are common targets of clinical trials for PD, Lewy Body Dementia and Multiple System Atrophy, few trials are currently targeting synuclein proteins to screen patients or to improve outcomes in AD (https://clinicaltrials.gov/).

Prior work from our group demonstrated that constitutive *SNCA* ablation in male J20 APP transgenic mice, a commonly used model of AD, alleviated memory deficits [25]. In this study, we used a murine ASO^*SNCA*^ to demonstrate that transient knockdown of αSyn could rescue cognition. We showed that the resulting lowering of αSyn rescued cognitive deficits in male APP mice, consistent with our previous results [25]. Thus, the combined results from ASO^*SNCA*^-injected APP mice and from APP/αSyn-KO mice indicate to us that ASO^*SNCA*^ might be a viable treatment option for men with AD-associated cognitive deficits.

We readily acknowledge that the results presented here relied on the use of transgenic APP mice. We previously reported that our J20 mice overexpress approximately threefold more APP than NTG mice [25, 29, 30, 39], a finding recently validated [23]. Consequently, we cannot rule out the possibility that the memory impairments seen in J20 mice are due to human APP overexpression. However, newer studies have demonstrated that behavioral and neural network abnormalities in J20 APP transgenic mice resemble those of *APP* knock-in mice [23], thus indicating that familial AD-linked APP mutations cause spatial learning and memory deficits in absence of APP overexpression. This said, future studies using *APP* knock-in animals [50] will be needed to address whether the gene deletion of *SNCA* or ASO^*SNCA*^ treatment can also rescue memory deficits in these models.

### Differential role of αSyn in learning and memory based on sex

The cognitive rescue caused by the murine ASO^*SNCA*^ treatment was only seen in male APP animals. Surprisingly, NTG female mice performed worse on spatial reference memory tasks when injected with ASO^*SNCA*^, while treated female APP mice showed no cognitive amelioration. To further demonstrate a differential role of αSyn across sexes in spatial learning and memory, we next determined whether female mice would be impaired if *SNCA* was constitutively ablated. As reported previously [9, 25], αSyn-KO males performed no differently than age-matched non-littermate NTG mice on hippocampal-dependent spatial reference memory tasks. Female αSyn-KO mice, however, displayed learning and retention memory impairments compared to both αSyn-KO males and NTG females. To our knowledge, these findings are the first to document a sex effect in response to *SNCA* knockdown or ablation, demonstrating that αSyn function differs in male and female brains.

Abeliovich et al. [1] originally described *SNCA*-KO male and female mice on a mixed 129/SvJxC57BL/6 genetic background. They found no differences between male and female mice on the open-field locomotor testing paradigm, but did not assess spatial memory. In addition, these same authors reported no differences in CA1 LTP between *SNCA*-KO and wild-type slices. However, the sex of the animals used in those electrophysiology experiments was not documented, making comparison to our data impossible. Another group reported no differences in hippocampal synaptic plasticity in αSyn and β-synuclein (βSyn) single KO mice as well as αSyn/βSyn double KO animals [10]. Their results using exclusively male mice are consistent with the lack of LTP and spatial learning deficits observed in our current and past studies. It is possible that the effects of sex documented here were masked in other work by the absence of dedicated groups to study sex as a key biological variable, as recently highlighted [37].

Despite the recurring evidence that sex can impact disease pathogenesis [31, 59], prior studies utilizing ASOs targeting *SNCA* often used mice of a single sex, when sex is reported at all, or did not examine data for possible sex effects [2, 7, 12, 42, 58, 63]. Lack of analysis and reporting could obscure important differences and delay the development of clinical therapies [37]. To our knowledge, our report is the first observation demonstrating that genetic ablation of *SNCA* differentially affects the cognition of male and female mice. Thus, reports of the beneficial effects of αSyn reduction based on experiments using exclusively male animals should not be assumed to apply similarly to females.

### EGR1 as a key mediator of spatial memory

There is overwhelming evidence demonstrating that *EGR1*-mediated transcriptional mechanisms play critical roles in neuronal plasticity, memory formation and consolidation (see for review [61]). These functions appear to fundamentally depend on multiple factors including: 1) a complex relationship between *EGR1* expression dosage, 2) the temporal expression of *EGR1* (e.g. acute vs. sustained expression) [21], 3) the type of memory process involved [53], and 4) the underlying brain regions [6, 24, 43, 61]. For these reasons, it has been proposed that *EGR1* can induce beneficial or deleterious effects [64] and that the outcome may depend on the target genes downstream of *EGR1* [61]. There is also existing evidence that *EGR1* can have sex-specific effects on behavior [15, 57] and that mouse strain can influence the expression of *EGR1* [44].

Several studies have documented an upregulation of *EGR1* at transcriptional and post-translational levels in AD brain tissue and in mouse models of the disease [4, 19, 20, 26, 33, 34]. Briefly, microarray analyses revealed that hippocampal *EGR1* transcriptional expression increased with AD severity [20]. Subsequent studies reported Egr1 protein elevations in cognitively-impaired Tg2576, APP/PS1 and 3xTg-AD mouse models of AD [4, 19, 26]. Conversely, knockdown of *EGR1* improved cognitive deficits in 3xTgAD mice [46]. Overall, these studies suggest that sustained elevated expression of *EGR1* is detrimental for long-term memory.

In our own studies, transcriptional profiling analyses revealed an increase in EGR1 transcripts in the hippocampi of female αSyn-KO mice compared to male littermates. We confirmed the elevation of *EGR1* gene products in the CA1 and DG regions of female αSyn-KO mice, regions known to play a critical role in long-term memory consolidation and retrieval (see [65] for review). Considering that spatial reference memory was impaired in female αSyn-KO mice and that electrophysiological recordings indicated a failure of female αSyn-KO mice to maintain LTP at CA1 SC synapses, these findings are in agreement with prior observations and indicate that sustained increased Egr1 may be deleterious for long-term memory.

Environmental interactions and neuronal activity are modulators of immediate early genes, such as *EGR1*. In turn, *ERG1* mediates many genes associated with neuronal signaling, protein translation and protein degradation [16]. While the results of our studies provide evidence for a novel connection between αSyn and Egr1, the molecular mechanism by which *SNCA* ablation impacts *EGR1* expression remains to be elucidated. However, our data unequivocally indicate that future studies should also carefully consider the effect of sex while investigating these mechanisms.

## Conclusions

To conclude, the translational and genetic results presented here revealed a gender-specific responsiveness to murine ASO^*SNCA*^ and a novel sex-specific role of αSyn on learning and memory in mice. By extension, these findings suggest this new role for αSyn should be considered when developing any *SNCA* modulating therapy. Finally and more broadly, these observations underscore the fundamental importance of including both sexes in experimental groups when performing studies.

### Supplementary Information

**Additional file 1**: Summary table of statistical tests and outcomes.

**Additional file 2**: Supplementary figures.

## Data Availability

All data needed to evaluate the conclusions in the paper are present in the paper and the Supplementary Materials, Additional files 1 and 2. The data can be provided following scientific review and a completed material transfer agreement. Requests for data and materials should be submitted to the corresponding author.

## Abbreviations

AD: Alzheimer’s disease
APP: J20 APP transgenic mice
ASO: Antisense oligonucleotide
Aβ: Amyloid-beta
BCM: Barnes circular maze
NTG: Non-transgenic mice
EGR1: Early growth response 1
WT: Wild-type
αSyn: Alpha-synuclein
αSyn-KO: *SNCA*-Null mice
