# Correction: *SNCA* genetic lowering reveals differential cognitive function of alpha-synuclein dependent on sex

**DOI:** 10.1186/s40478-024-01789-w

**Published:** 2024-06-11

**Authors:** Jennifer L. Brown, Damyan W. Hart, Gabriel E. Boyle, Taylor G. Brown, Michael LaCroix, Andrés M. Baraibar, Ross Pelzel, Minwoo Kim, Mathew A. Sherman, Samuel Boes, Michelle Sung, Tracy Cole, Michael K. Lee, Alfonso Araque, Sylvain E. Lesné

**Affiliations:** 1 Graduate Program in Neuroscience, University of Minnesota, Minneapolis, MN USA; 2 Department of Neuroscience, University of Minnesota, Minneapolis, MN USA; 3 Medical Scientist Training Program, University of Minnesota, Minneapolis, MN USA; 4 Institute for Translational Neuroscience, University of Minnesota, Wallin Medical Biosciences Building (Room 4-114), 2101 Sixth Street SE, CDC 2641, Minneapolis, MN 55414 USA; 5 Ionis Pharmaceuticals Inc., Carlsbad, CA USA; 6 Present Address: Medical Scientist Training Program, University of Texas Southwestern Medical School, Dallas, TX 75390 USA; 7 Present Address: Graduate Program in Molecular and Cellular Biology, University of Washington, Seattle, WA 98195 USA; 8 Present Address: Department of Neurosciences, University of the Basque Country UPV/EHU, Leioa, Spain; 9 Present Address: Bloomberg School of Public Health, Johns Hopkins University, Baltimore, MD 21218 USA; 10 Present Address: n-Lorem Foundation, Carlsbad, CA 92010 USA

**Correction: Acta Neuropathol Commun (2022) 10:180** 10.1186/s40478-022-01480-y

Following the publication of the original article [1], it was noted that due to a technical error Fig. 1c was incorrect. The images presented in Fig. 1C for the “2 weeks ASO” and “3 weeks ASO” groups are identical. Unfortunately, this was not detected prior to publication. The authors have reviewed the original images and raw data files for this experiment and they can confirm that the published images for the “2 week ASO” group are not consistent with the original raw files but depict the raw images corresponding to the "3 weeks ASO” group. The correct figure is given hereafter.

The correction does not change the conclusions of the published work.

The incorrect Fig. 1(C) reads:

**Fig. 1:**
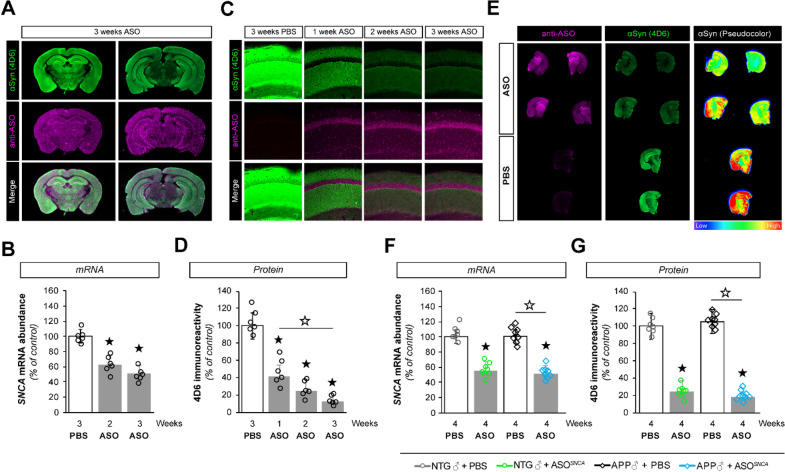
ASO1 disperses throughout the brain and lowers *SNCA* gene expression. **A** Infra-red imaging documenting the widespread distribution of ASOs 3 weeks after injection using anti- ASO (pink) and anti-αSyn (4D6, green) antibodies. **B** Reduction in *SNCA* mRNA at 2 and 3 weeks post-injection as determined by RT-qPCR. **C** Confocal imaging illustrating the presence of ASO1 (pink) and a corresponding decrease in αSyn (green) in mouse hippocampi. **D** Quantification of hippocampal 4D6 immunoreactivity in ASO1 or PBS treated mice. **E** Infra-red imaging detectedαSyn (green) and ASO (pink) in coronal brain sections from PBS and ASO treated mice. The relative αSyn signal was lower in ASO-injected animals than in PBS-injected animals (pseudocolor). **F**, **G** Measurements of *SNCA* mRNA abundance by RT-qPCR (**F**) and αSyn protein amounts by immunofluorescence (**G**) in transgenic (APP) and non-transgenic (NTG) animals treated with PBS or ASO. Histogram bars represent mean ± SEM. ★P < 0.05 compared to NTG + PBS, ☆P < 0.05 compared to APP + PBS

The correct Fig. 1(C) should read:

**Fig. 1:**
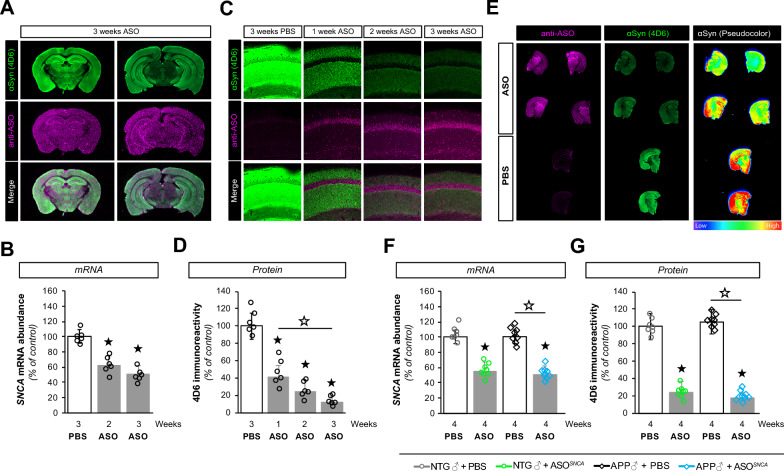
ASO1 disperses throughout the brain and lowers *SNCA* gene expression. **A** Infra-red imaging documenting the widespread distribution of ASOs 3 weeks after injection using anti- ASO (pink) and anti-αSyn (4D6, green) antibodies. **B** Reduction in *SNCA* mRNA at 2 and 3 weeks post-injection as determined by RT-qPCR. **C** Confocal imaging illustrating the presence of ASO1 (pink) and a corresponding decrease in αSyn (green) in mouse hippocampi. **D** Quantification of hippocampal 4D6 immunoreactivity in ASO1 or PBS treated mice. **E** Infra-red imaging detectedαSyn (green) and ASO (pink) in coronal brain sections from PBS and ASO treated mice. The relative αSyn signal was lower in ASO-injected animals than in PBS-injected animals (pseudocolor). **F**, **G** Measurements of *SNCA* mRNA abundance by RT-qPCR (**F**) and αSyn protein amounts by immunofluorescence (**G**) in transgenic (APP) and non-transgenic (NTG) animals treated with PBS or ASO. Histogram bars represent mean ± SEM. ★P < 0.05 compared to NTG + PBS, ☆P < 0.05 compared to APP + PBS

The correct figure has been included in this correction, and the original article [1] has been corrected.
