# Supplementary material for: SNCA genetic lowering reveals differential cognitive function of alpha-synuclein dependent on sex
Source: Acta Neuropathol Commun. 2022 Dec 14;10:180. doi: 10.1186/s40478-022-01480-y (PMC9749314; doi:10.1186/s40478-022-01480-y)
Supplement: Supplementary file 1 — Additional file 1: Summary table of statistical tests and outcomes. [file 40478_2022_1480_MOESM1_ESM.docx]

Additional File 1

Supplementary Materials for

***SNCA* genetic lowering reveals differential cognitive function of alpha-synuclein dependent on sex**

Jennifer L. Brown^1,2,4^, Damyan W. Hart^2,4^, Gabriel S. Boyle^2,4,§^, Taylor G. Brown^2,3,4^, Michael LaCroix^2,4,$^, Andres Baraibar^2^, Ross Pelzel^1,2,4^, Minwoo Kim^2,4^, Mathew A. Sherman^2,4^, Sam Boes^2,4^, Michelle Sung^2,4^, Tracy Cole^5^, Alfonso Araque^1,2^, Michael K. Lee^2,4^, Holly Kordasiewicz^5^ & Sylvain E. Lesné^1,2,4*^

*Correspondence:

Sylvain E. Lesné, Ph.D.

University of Minnesota

Wallin Medical Biosciences Building (Room 4-114)

2101 Sixth Street SE,

CDC 2641

55414 Minneapolis, MN

Office: (612)-626-8341

Fax: (612)-626-5009

email: [lesne002@umn.edu](mailto:lesne002@umn.edu)

website: <https://lesnelab.org>

**This file includes:**

Figs. S1-S8

**
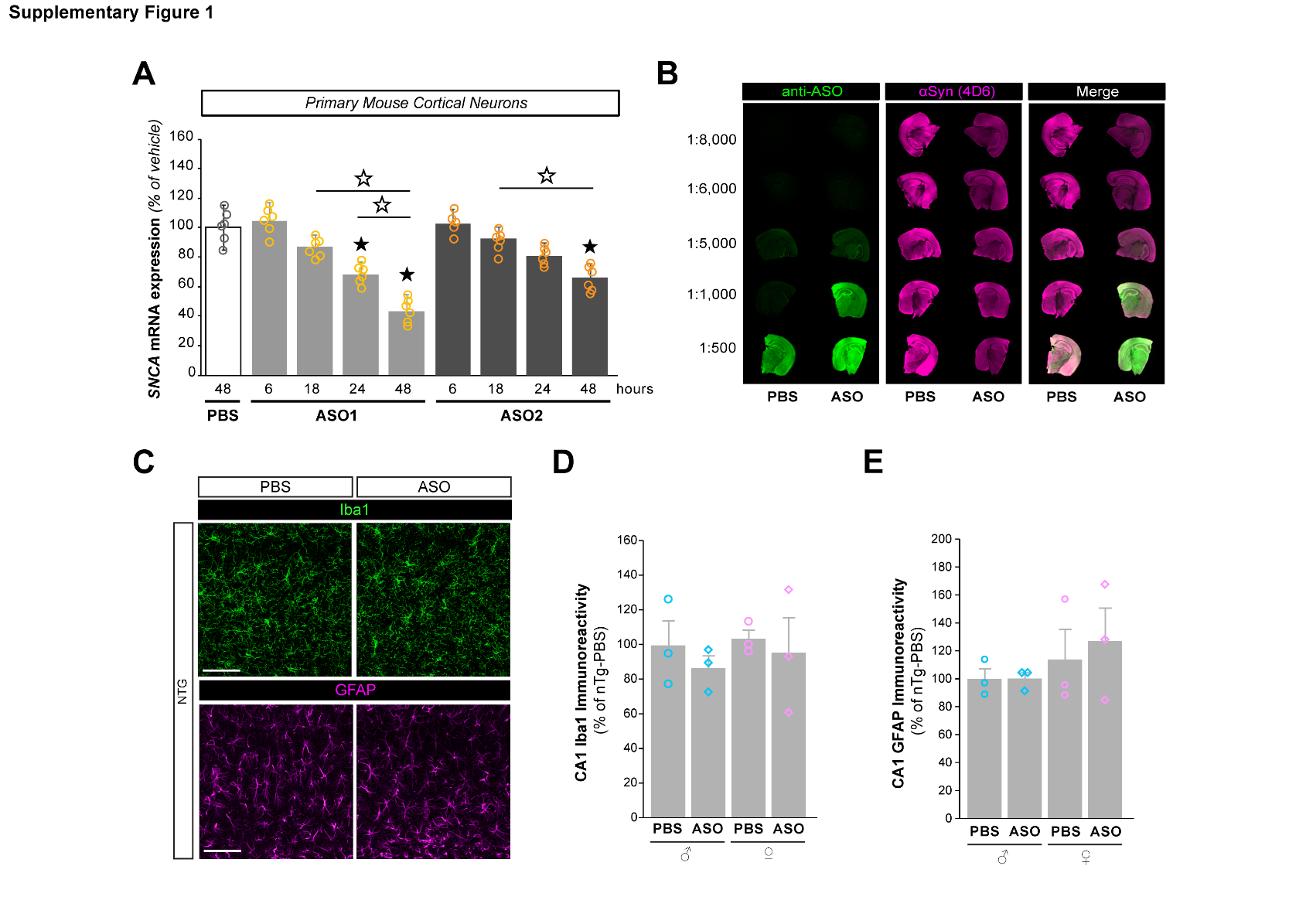
**

**Supplementary Figure S1.** **ASO1 decreases *SNCA* mRNA without overt inflammation.** (**A**) *SNCA* mRNA expression in cortical neurons treated with PBS or 300 µg of ASO1 or ASO2 over 48 hours was determined by RT-qPCR. Actin was used to normalize the data. (**B**) Anti-ASO antibody was tested at different concentrations on both ASO and PBS-injected mouse brains. A concentration of 1:1000 strongly labeled the ASO-injected brain while not labeling the PBS slice. Staining with 4D6 for αSyn at 1:250 labeled PBS brains more strongly than ASO brains. Merging the anti-ASO and 4D6 images demonstrates that decreases in αSyn are detectable via infra-red imaging (LiCor). (**C**) Representative confocal images of hippocampal sections of non-transgenic C57BL/6J mouse brains, injected with PBS or ASO1, immunolabelled with the antibodies Iba1 (green) and GFAP (pink). (**D**, **E**) Density of Iba1 (*D*) and GFAP (*E*) immunoreactivity in C57BL/6J mice of both sexes, following injection with PBS or ASO1. Data represent mean ± SEM; ★P < 0.05 compared to PBS, ☆P < 0.05 compared to earlier time points.

**
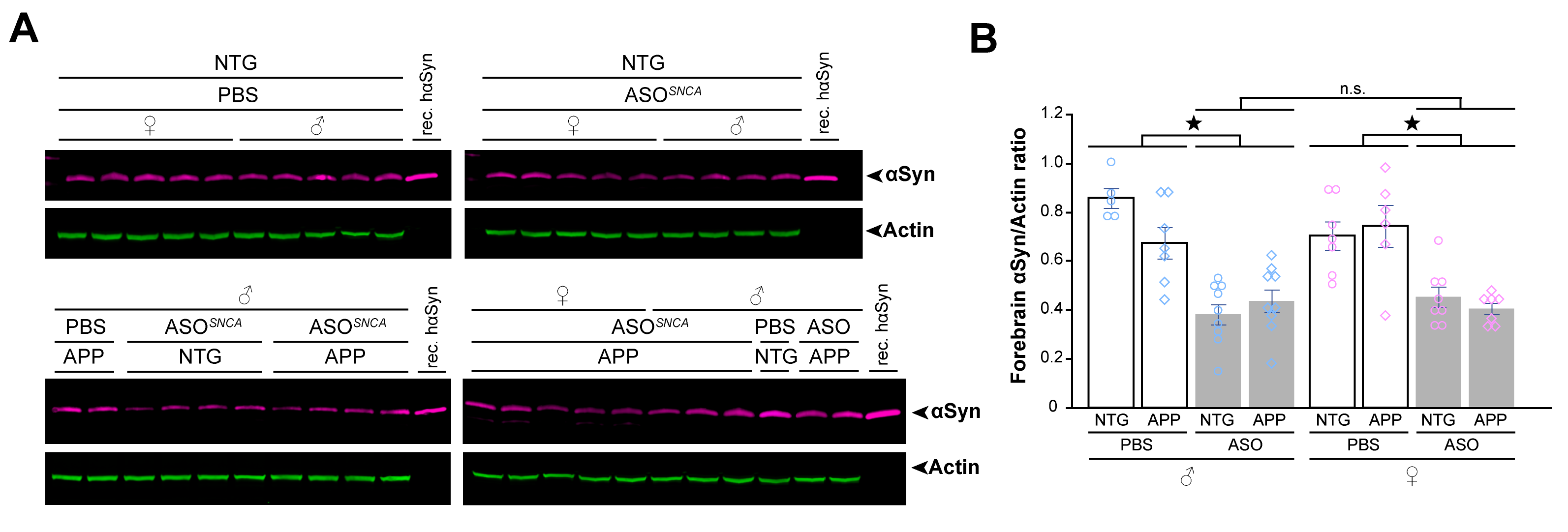
**

**Supplementary Figure S2. ɑSyn expression in ASO- and PBS-injected mice.**

(**A**) Representative western blots of forebrain lysates from both male and female mice injected with either PBS or ASO1 stained for αSyn (4D6; magenta) and actin (anti-actin; green). Recombinant human αSyn was used as a positive control. (**B**) Quantification of αSyn relative to actin showed the expected decrease in response to ASO1 injection, but no differences in αSyn expression between female and male mice or between genotypes. Data represent mean ± SEM. Three-way RM-ANOVA followed by Tukey HSD, F_(7,57)_ = 11.6953, *P* < 0.0001. ^★^P < 0.05 compared to PBS.

**
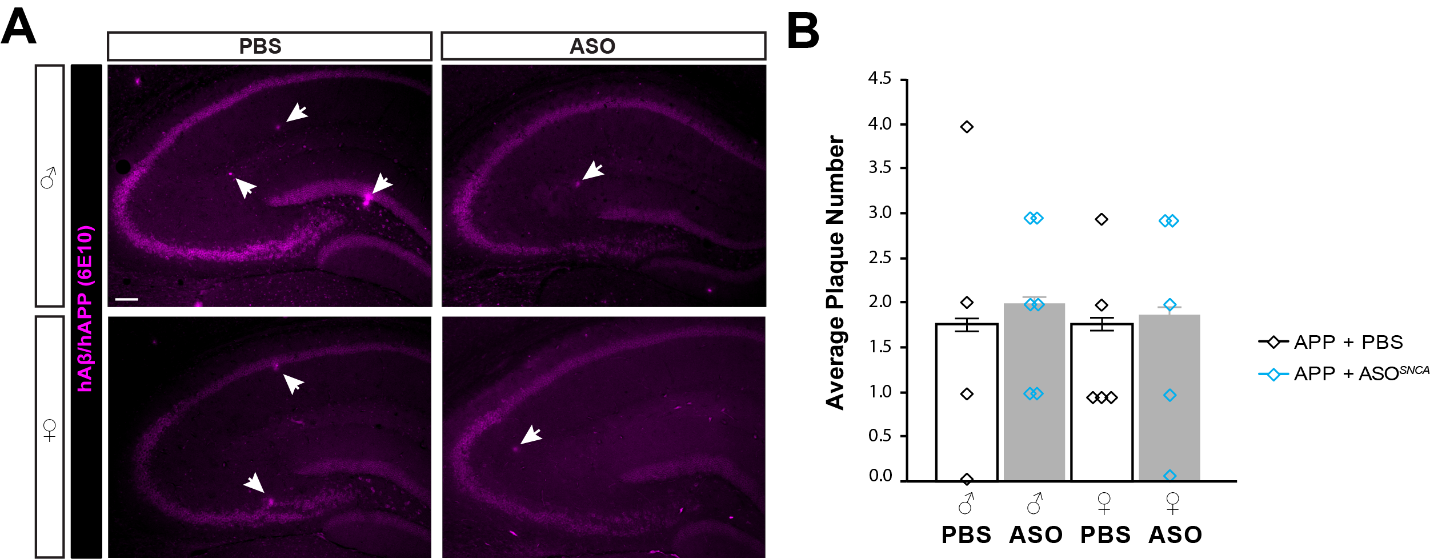
**

**Supplementary Figure S3. Amyloid plaque burden in PBS and ASO-injected APP mice.** (**A**) Representative confocal images of hippocampal sections of J20 APP mouse brains, injected with PBS or ASO, immunolabelled with the antibody 6E10, detecting human Aβ1-16. White arrows indicate Aβ plaques. Images were taken at 20x; scale bar = 100 µm. (**B**) Amyloid-beta plaques were quantified using unbiased stereology. There were no differences in plaque number between any groups. Data represent mean ± SEM; Two-way ANOVA followed by Tukey HSD, *F*_(3,20)_ = 0.1055, *P* = 0.9557.

**
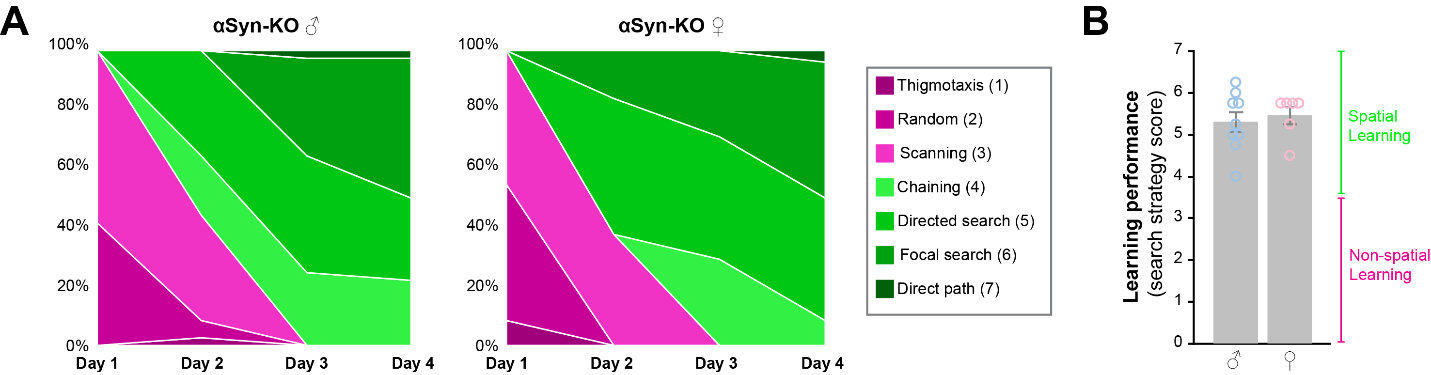
**

**Supplementary Fig. S4. Spatial learning strategies are similar between groups.** During learning, strategies on each trial were categorized as either non-spatial (1-3) or spatial (4-7). (**A**) Relative distribution of the different search strategies used by male ɑSyn-KO (left) and female ɑSyn-KO (right) mice over four Barnes circular maze test days. (**B**) Learning performance: mean search strategy scores on day 4 of Barnes circular maze testing. The average search strategy score during learning was not different between male and female animals. Data represent mean ± SEM. Student *t* test, *P* = 0.6565; (n = 8 male, n = 6 female).

**
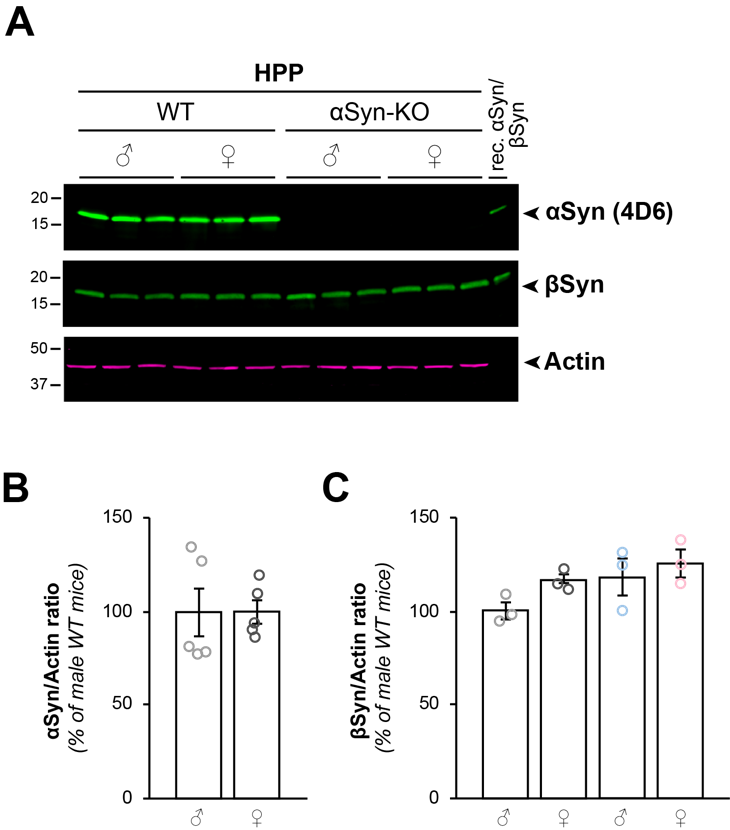
**

**Supplementary Fig. S5. Protein abundance of αSyn and βSyn in the hippocampus of female and male WT and ɑSyn-KO mice.** (**A**) Representative western blot of hippocampal lysates from both male and female mice stained for αSyn (4D6; green) and actin (anti-actin; magenta). Lysates from αSyn-KO mice were used as negative controls, recombinant αSyn and βSyn were used as a positive control. (**B, C**) Quantification of αSyn *(B)* and βSyn *(C)* relative to actin showed no difference in αSyn or βSyn expression between female and male WT mice, nor between genotypes. Data represent mean ± SEM. Student t test, *P* = 0.8371 and two-way ANOVA followed by Tukey HSD, *F*_(3,8)_ = 2.5754, *P* = 0.1266 respectively.

**
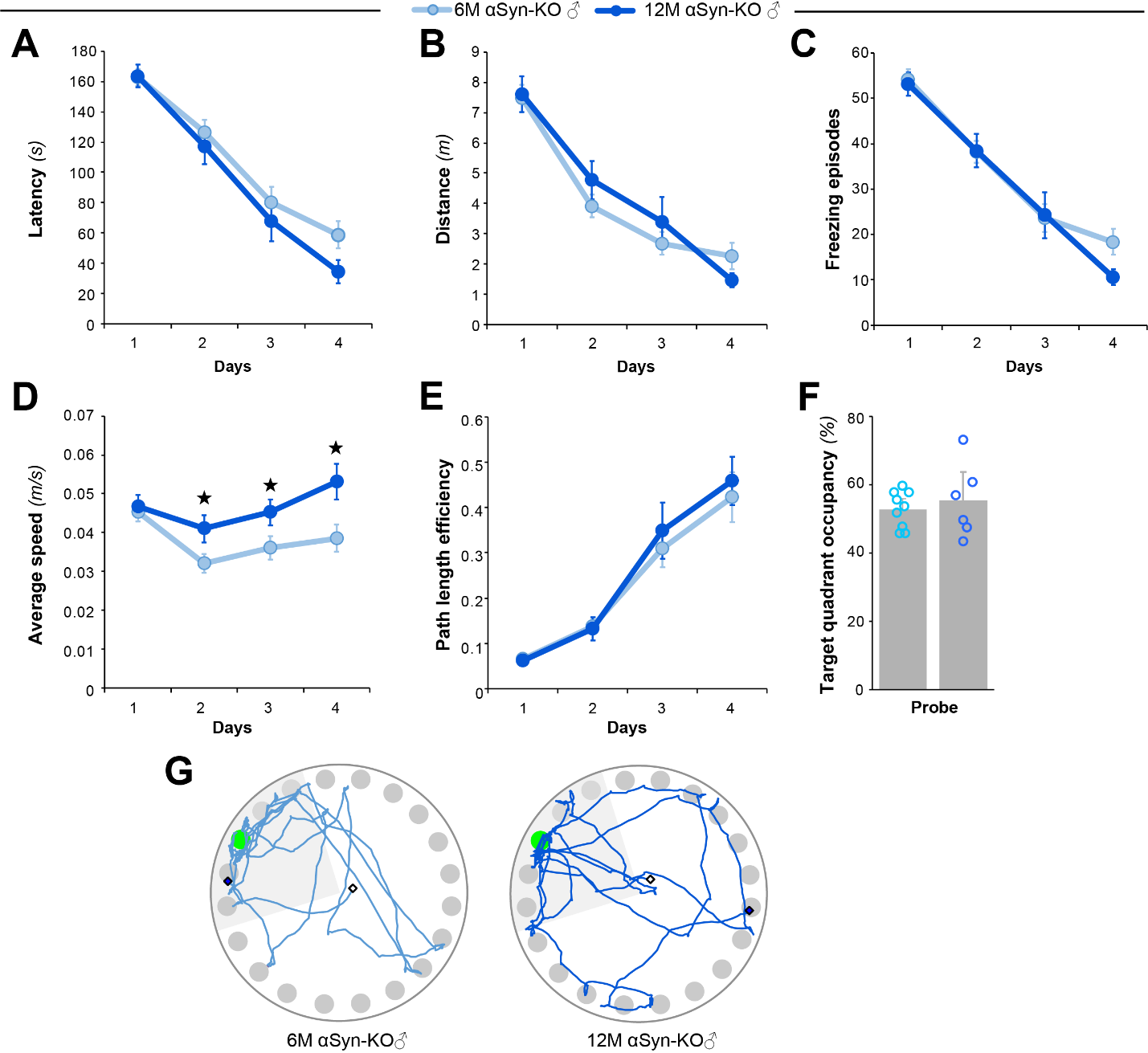
**

**Supplementary Fig. S6. Effect of age on spatial learning and memory in male αSyn-KO mice.** There were no differences between 6-month and 12-month-old male αSyn-KO mice on latency (**A**), distance traveled (**B**), or freezing episodes (**C**). Older mice displayed higher average speeds than 6-month-old mice (two-way ANOVA, F_(7,239)_ = 4.5302, *P* < 0.0001 with effects of Day, [*P* = 0.0081] and Age [*P* = 0.0002] but no Age*Day interaction [P = 0.2405]) (**D**). There were no differences in path efficiency (**E**) or target quadrant occupancy (**F**) between groups. Representative track plots, with escape box location indicated by a green circle (**G**). Bars represent mean ± SEM in panels A-F. ★*P* < 0.05 compared to 6M mice, *n*= 6-8 mice/age.

**
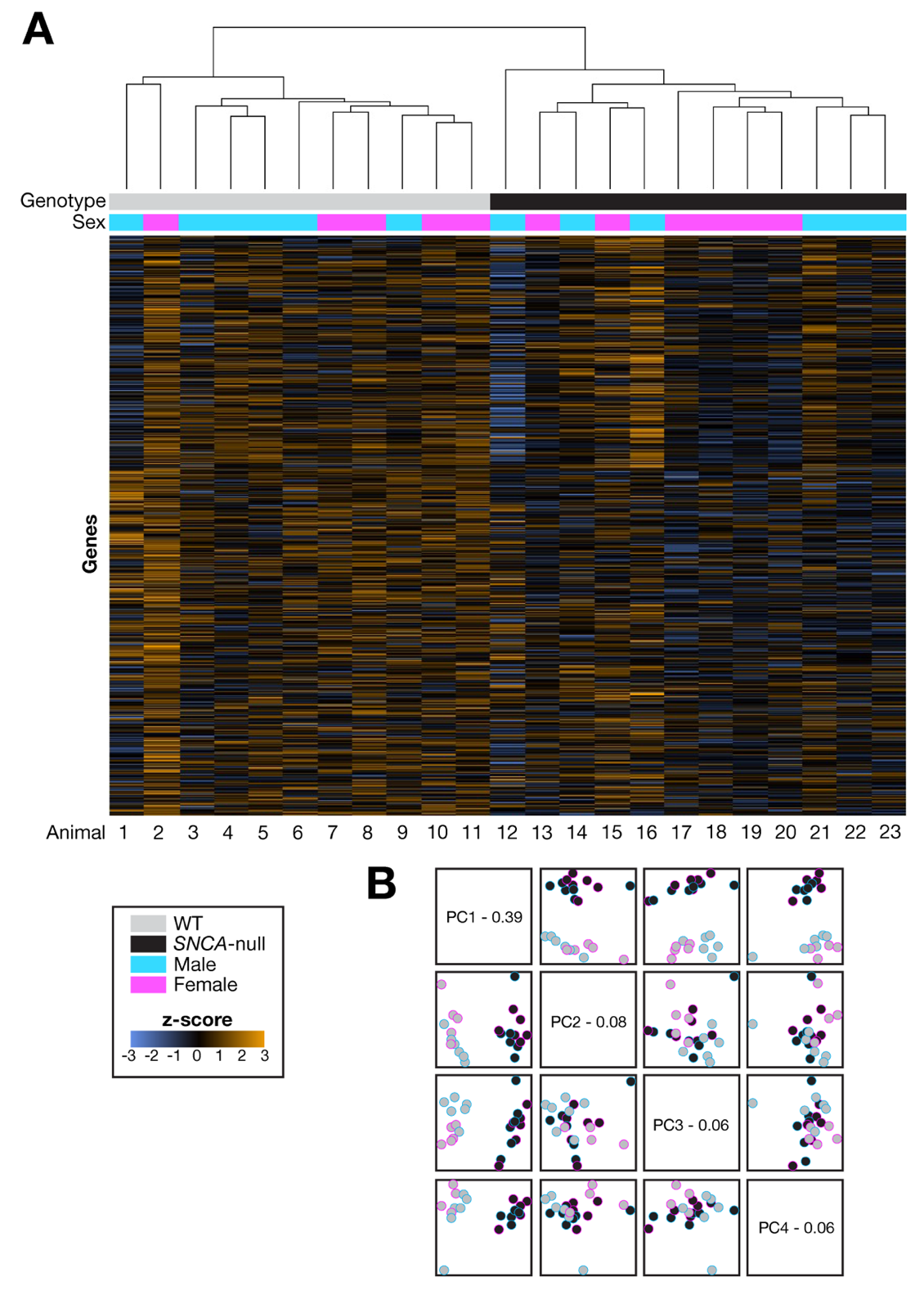
**

**Supplementary Fig. S7. Unsupervised hierarchical clustering shows clear separation by genotype.** (**A**) Normalized z-scores for each gene, for each animal, were grouped using unsupervised hierarchical clustering. (**B**) Principal component analysis, where PC1 (39%) distinguishes samples by genotype.

**
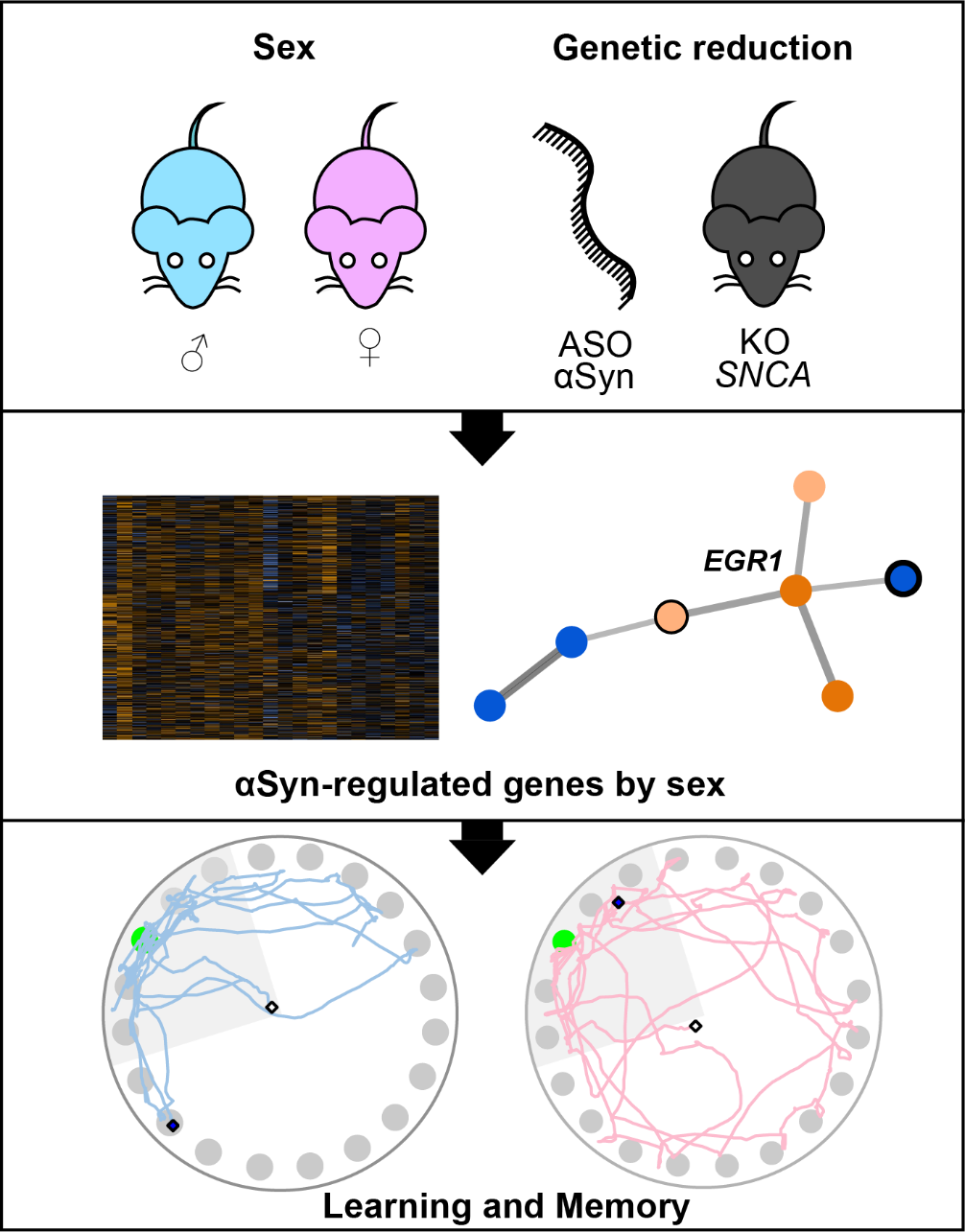
**

**Supplementary Fig. S8. Graphical summary.** Reduction of *SNCA* gene transcripts in mice reveled sex-specific differences in spatial learning and memory, as well as a differentially expressed gene network centered around *EGR1*.
